# Combining anodic alcohol oxidative coupling for C–C bond formation with cathodic ammonia production

**DOI:** 10.1093/nsr/nwae134

**Published:** 2024-04-04

**Authors:** Leitao Xu, Wei Chen, Cairong Wang, Wenjie Wu, Yelin Yao, Zhifeng Huang, Jingcheng Wu, Ming Yang, Yandong Wu, Dianke Xie, Yuqin Zou, Shuangyin Wang

**Affiliations:** State Key Laboratory of Chemo/Bio-Sensing and Chemometrics, College of Chemistry and Chemical Engineering, Advanced Catalytic Engineering Research Center of the Ministry of Education, Hunan University, Changsha 410082, China; State Key Laboratory of Chemo/Bio-Sensing and Chemometrics, College of Chemistry and Chemical Engineering, Advanced Catalytic Engineering Research Center of the Ministry of Education, Hunan University, Changsha 410082, China; State Key Laboratory of Chemo/Bio-Sensing and Chemometrics, College of Chemistry and Chemical Engineering, Advanced Catalytic Engineering Research Center of the Ministry of Education, Hunan University, Changsha 410082, China; State Key Laboratory of Chemo/Bio-Sensing and Chemometrics, College of Chemistry and Chemical Engineering, Advanced Catalytic Engineering Research Center of the Ministry of Education, Hunan University, Changsha 410082, China; State Key Laboratory of Chemo/Bio-Sensing and Chemometrics, College of Chemistry and Chemical Engineering, Advanced Catalytic Engineering Research Center of the Ministry of Education, Hunan University, Changsha 410082, China; State Key Laboratory of Chemo/Bio-Sensing and Chemometrics, College of Chemistry and Chemical Engineering, Advanced Catalytic Engineering Research Center of the Ministry of Education, Hunan University, Changsha 410082, China; State Key Laboratory of Chemo/Bio-Sensing and Chemometrics, College of Chemistry and Chemical Engineering, Advanced Catalytic Engineering Research Center of the Ministry of Education, Hunan University, Changsha 410082, China; State Key Laboratory of Chemo/Bio-Sensing and Chemometrics, College of Chemistry and Chemical Engineering, Advanced Catalytic Engineering Research Center of the Ministry of Education, Hunan University, Changsha 410082, China; State Key Laboratory of Chemo/Bio-Sensing and Chemometrics, College of Chemistry and Chemical Engineering, Advanced Catalytic Engineering Research Center of the Ministry of Education, Hunan University, Changsha 410082, China; State Key Laboratory of Chemo/Bio-Sensing and Chemometrics, College of Chemistry and Chemical Engineering, Advanced Catalytic Engineering Research Center of the Ministry of Education, Hunan University, Changsha 410082, China; State Key Laboratory of Chemo/Bio-Sensing and Chemometrics, College of Chemistry and Chemical Engineering, Advanced Catalytic Engineering Research Center of the Ministry of Education, Hunan University, Changsha 410082, China; State Key Laboratory of Chemo/Bio-Sensing and Chemometrics, College of Chemistry and Chemical Engineering, Advanced Catalytic Engineering Research Center of the Ministry of Education, Hunan University, Changsha 410082, China

**Keywords:** anodic alcohol oxidative coupling, C–C bond formation, cinnamaldehyde, cathodic ammonia production

## Abstract

Electrocatalytic oxidation of alcohols using heterogeneous catalysts is a promising aqueous, energy-efficient and environmentally friendly approach, especially for coupling different alcohols to prolong the carbon chain via co-oxidation. Precisely regulating critical steps to tailor electrode materials and electrolyte composition is key to selectively coupling alcohols for targeted synthesis. However, selectively coupling different alcohols remains challenging due to the lack of effective catalyst and electrolyte design promoting specific pathways. Herein, we demonstrate a paired electrolysis strategy for combining anodic oxidative coupling of ethanol (EtOH) and benzyl alcohol (PhCH_2_OH) to synthesize cinnamaldehyde (CAL) and cathodic ammonia production. The strategies involve: (i) utilizing the salt-out effect to balance selective oxidation and coupling rates; (ii) developing platinum-loaded nickel hydroxide electrocatalysts to accelerate intermediate coupling kinetics; (iii) introducing thermodynamically favorable nitrate reduction at the cathode to improve coupling selectivity by avoiding hydrogenation of products while generating valuable ammonia instead of hydrogen. We achieved 85% coupling selectivity and 278 μmol/h NH_3_ productive rate at 100 mA/cm^2^ with a low energy input (∼1.63 V). The membrane-free, low energy, scalable approach with a wide substrate scope highlights promising applications of this methodology. This work advances heterogeneous electrocatalytic synthesis through rational design principles that integrate anodic oxidative coupling with cathodic nitrate reduction reactions, having synergistic effects on efficiency and selectivity.

## Teaser

Integrating anodic oxidative coupling with cathodic nitrate reduction reactions enabled efficient electrocatalytic co-oxidative coupling of ethanol and benzyl alcohol to produce cinnamaldehyde.

## INTRODUCTION

The utilization of renewable energy for the synthesis of value-added chemicals will be a significant part of the future development of sustainability [1,2]. Electrochemical synthesis has gained momentum as a green alternative for establishing environmentally friendly and sustainable processes in recent years, since it takes advantage of clean electrons to replace dangerous and toxic redox reagents [3–6]. The past decade has witnessed the rapid development of numerous electrochemical reactions for synthesizing various important molecules [7–10], such as a coupling reaction that can directly link two different molecules together and allow for a high atomic economy. However, most established electrochemical coupling reactions use low-conductivity organic solvents, resulting in slow kinetics. Additionally, organic electrolytes lead to complex post-treatment and high separation costs [11,12].

Water is an abundant resource that can be split by renewable energy to generate active hydrogen and oxygen species, which are often reactive intermediates in catalytic reactions [13,14]. Utilizing these renewable H/O species to reduce or oxidize organics for value-added chemicals is a promising green route due to its low energy input, avoidance of chemical reagents and mild conditions [15,16]. Electrocatalytic conversion in aqueous solutions has gained widespread interest, as it is a highly efficient route for oxidizing alcohols, aldehydes and amines into value-added products [17–20]. Alcohol oxidation is a primary focus of energy and chemical conversion, with potential applications in biomass utilization and fine-chemical synthesis [21–24]. Ni-based electrocatalysts are promising candidates, as the multivalent Ni can facilitate organic oxidation [25–28]. The alcohol oxidation reaction on Ni-based electrocatalysts involves many intermediate species, such as *RCHOH, *RCHO, *RCH(OH)_2_ and *RC(OH)_2_ [29,30]. However, a single acid product is usually the result, since intermediates with faster kinetics dominate in most reported electrocatalytic alcohol oxidation reactions.

Aldehydes are highly useful synthetic intermediates that can undergo diverse reactions to form many products [31,32]. Coupling the key intermediate *RCHO from alcohol oxidation at the anode with other nucleophilic species allows the synthesis of high-value chemicals [33]. For example, two identical or different aldehydes can produce *α,β*-unsaturated aldehydes by aldol condensation, which is widely used to make pharmaceutical and fine-chemical intermediates. Directly synthesizing such compounds from alcohols via co-oxidative coupling is more scientifically valuable and impactful than post-condensation approaches [34,35]. However, effective methods to achieve co-oxidative C=C bond formation between two alcohols remain elusive, limiting the utility of alcohol oxidation. In our previous work, the selective conversion of alcohol to aldehyde was achieved by reducing the alkalinity of the electrolyte and developing a salting-out strategy [36]. While this is encouraging, matching the selective oxidation rates between two alcohols and overcoming differences in key intermediate adsorption/activation remain challenges for electrocatalytic coupling to higher-value chemicals. Our aim is to develop an electrocatalytic co-oxidative coupling strategy to directly synthesize *α,β*-unsaturated aldehydes from alcohols, addressing a major challenge in synthetic chemistry. Advances in catalyst and electrolyte design are needed to realize the significant potential of co-oxidative alcohol coupling reactions.

**Figure 1.**
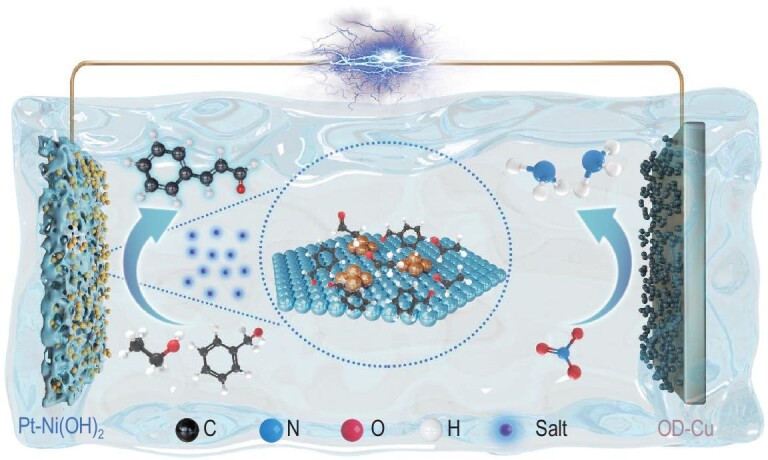
Schematic diagram for combining anodic alcohol oxidative coupling with NH_3_ production.

In this work, we report a novel paired electrolysis approach that integrates the synthesis of a value-added chemical with sustainable ammonia production (Fig. 1). Specifically, we develop a bifunctional electrolysis cell that couples the anodic oxidative coupling of ethanol (EtOH) and benzyl alcohol (PhCH_2_OH) to generate cinnamaldehyde (CAL) with the cathodic reduction of nitrate to ammonia. The integration of experiment, theoretical simulation and *in situ* electrochemical spectroscopy reveals that suppressing the over-oxidation of alcohols by the salt-out strategy and accelerating cross-coupling of key intermediates by developing efficient electrocatalysts, is vital for the coupling reaction. More importantly, the nitrate reduction reaction (NO_3_RR) was employed to construct a novel pair electrolysis system, which can not only produce more valuable NH_3_ at the cathode, but also effectively improve the selectivity of the coupling reaction by inhibiting the hydrogenation and reducing energy consumption. Finally, this strategy was demonstrated to be facile for scaled-up applications.

## RESULTS AND DISCUSSION

Nickel hydroxide (Ni(OH)_2_) is a versatile, inexpensive and efficient electrocatalyst for the electrochemical oxidation of alcohols. Ni(OH)O generated by applying an external potential to the Ni(OH)_2_ electrode is the active species that catalyzes the dehydrogenation of alcohols to aldehydes, ketones or acids (Fig. S1 in Supplementary Data). For the electrochemical co-oxidation coupling reaction of alcohols, Ni(OH)_2_ was chosen as the anodic electrocatalyst. It was synthesized by a hydrothermal method and characterized by X-ray diffraction (XRD), scanning electron microscopy (SEM) and X-ray photoelectron spectroscopy (XPS), confirming its structure, morphology and composition (Figs S2–S4). Ethanol (EtOH) and Benzyl alcohol (PhCH_2_OH) were selected as model substrates for the reaction, which are the precursors to the synthesis of CAL. The electro-oxidation of EtOH and PhCH_2_OH over the Ni(OH)_2_ was carried out. Linear sweep voltammetry (LSV) was first performed to evaluate the electrocatalytic oxidation of EtOH and PhCH_2_OH over the Ni(OH)_2_ electrocatalyst (Fig. S5). The oxidation current of PhCH_2_OH was larger than that of EtOH at an identical concentration, attributed to the lower bond dissociation free energy (BDFE) of PhCH_2_OH facilitating the reaction with Ni(OH)O. In accordance with our previous work, the bulk electrolysis experiment was conducted in 1.5 M K_2_CO_3_ with 100 mM PhCH_2_OH and 200 mM EtOH under 100 mA/cm^2^ current density in an undivided cell. The distribution of products was analyzed by gas chromatography-mass spectrometry and ^1^H-nuclear magnetic resonance (^1^H NMR) (Figs S6 and S7). However, no coupling product was observed after the reaction. Increasing EtOH concentration resulted in minor CAL, indicating selective alcohol oxidation alone does not enable cross-coupling (Fig. S8).

**Figure 2.**
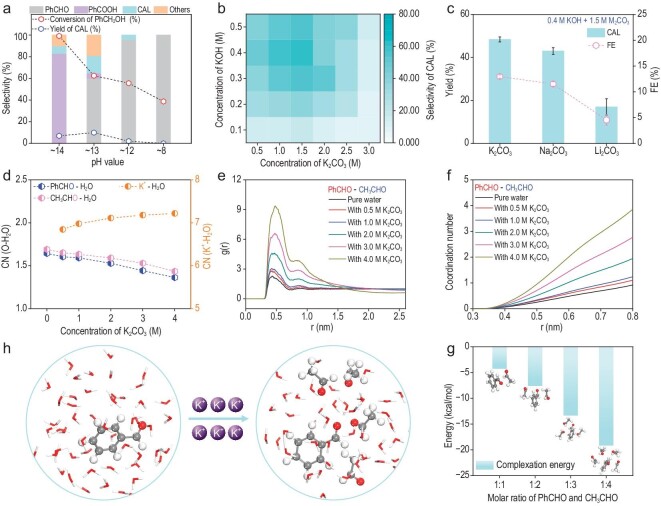
Anodic electro-oxidation coupling of EtOH and PhCH_2_OH to synthesize CAL over the Ni(OH)_2_ electrode. (a) The effect of pH values on the reaction. (b) The effect of K_2_CO_3_ concentration on the reaction. (c) The effect of cation types on the reaction. (d) The coordination number (CN) of O–H and K^+^–O as a function of cation concentration in the electrolyte with PhCHO and CH_3_CHO. (e) Radial distribution function (RDF) for the PhCHO and CH_3_CHO. (f) The coordination number curves for the PhCHO and CH_3_CHO. (g) The binding energy of PhCHO and CH_3_CHO under different molar ratios. (h) Illustration of the effect of K^+^ ion concentration on the reaction. Other reaction conditions for (a), (b) and (c): 100 mM PhCH_2_OH, 600 mM EtOH, Ni(OH)_2_ anode, Pt mesh cathode, 5 mL electrolyte, 100 mA/cm^2^, 2 h, room temperature.

The key for alcohol coupling is dehydration condensation between *in-situ*-generated PhCHO and CH_3_CHO intermediates. Density functional theory (DFT) calculations analyzed the thermodynamics of the non-electrochemical reaction to improve coupling efficiency. The deprotonation of CH_3_CHO was identified as the rate-determining step (RDS), with an energy barrier of 14.2 kcal/mol under alkaline conditions and 67.3 kcal/mol at neutral conditions. This implies that the RDS is strongly influenced by solution pH (Fig. S9). Based on the above analysis, we investigated the effects of pH on the electrochemical co-oxidation of PhCH_2_OH and EtOH (Fig. 2a). In the mixture of 0.1 M KOH and 0.45 M K_2_CO_3_ (pH ∼13), 63% conversion but only 16% CAL selectivity was observed, along with PhCOOH generation. This is attributed to the lower aldehyde hydration barrier at high alkalinity (Figs S10 and S11), as evidenced by NMR showing increased hydrated aldehyde content from 23% to 47% when increasing KOH from 0.01 M to 0.1 M (Figs S12 and S13). In 1 M KOH (pH ∼14), PhCOOH became the major product. Under high alkalinity, the faster kinetics of CH_3_CHO hydration (barrier 10.5 kcal/mol) dominate over slower CH_3_CHO deprotonation (barrier 14.2 kcal/mol), resulting in oxidation to CH_3_COOH rather than coupling (Figs S14 and S15).

Balancing alcohol over-oxidation and cross-coupling rates is key for efficient coupling reactions. Specifically, we believe that maintaining a certain alkalinity and cation concentration will achieve efficient coupling reactions. The effects of varying alkalinity (0.1–0.5 M KOH) and cation concentrations (0.5–3 M K_2_CO_3_) on the reaction were investigated (Fig. 2b and Figs S16 and S17). In mixed KOH/K_2_CO_3_ electrolytes, CAL selectivity increased with K_2_CO_3_ concentration from 0.5 to 1.5 M, then decreased above 1.5 M. The optimized 0.4 M KOH and 1.5 M K_2_CO_3_ electrolyte achieved 88% conversion and 55% CAL selectivity. Cations modulate hydration while alkalinity promotes deprotonation for cross-coupling. Evaluating other alkali metal ions showed slightly lower CAL selectivity with Na_2_CO_3_ and Li_2_CO_3_ versus K_2_CO_3_ (Fig. 2c).

Molecular dynamics (MD) simulations provided insights into the effect of cation concentration on reaction kinetics. With increasing K_2_CO_3_, aldehydes transitioned from disordered dispersion to aggregation (Figs S18 and S19). Radial distribution function (RDF) and integrated RDF showed lower H_2_O density around aldehydes but higher H_2_O density around K^+^ in concentrated electrolytes (Figs S20–S22). The coordination number (CN) of aldehydes decreased while the CN of K^+^ increased with higher K_2_CO_3_ (Fig. 2d). Additionally, fewer aldehyde-H_2_O hydrogen bonds (HBs) but more aldehyde-aldehyde HBs occurred at higher cation levels (Fig. S23). This indicates that cations disrupt aldehyde–H_2_O HBs but enhance aldehyde–aldehyde interactions, causing aggregation. RDF results also showed increased CH_3_CHO density and CN around PhCHO at higher K_2_CO_3_ concentrations (Fig. 2e and f). DFT revealed larger PhCHO–CH_3_CHO binding energy with more surrounding CH_3_CHO (Fig. 2g). In summary, cations induce aldehyde aggregation, increasing local CH_3_CHO concentration around PhCHO to kinetically favor coupling (Fig. 2h). Precisely modulating the cation concentration is thus critical for activating intermediates for co-oxidative electrosynthesis.

**Figure 3.**
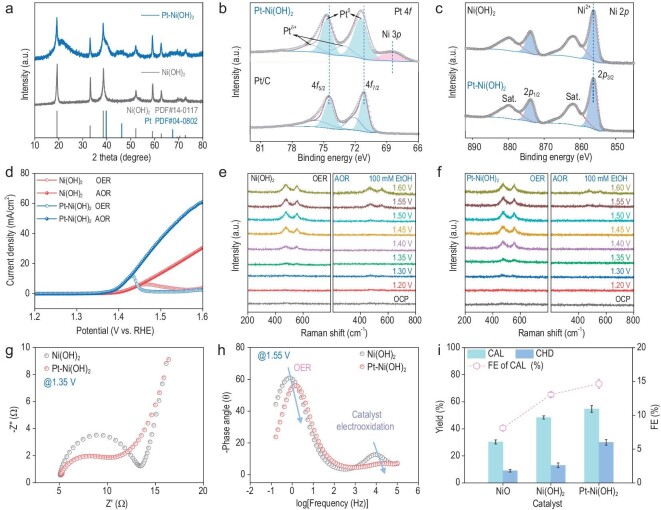
Developing the anode electrocatalyst enhances coupling efficiency. (a) XRD of Ni(OH)_2_ and Pt-Ni(OH)_2_. (b) XPS spectrum of the Pt 4f region of Pt-Ni(OH)_2_ and Pt/C. (c) XPS spectrum of the Ni 2p region of Ni(OH)_2_ and Pt-Ni(OH)_2_. (d) LSV (10 mV/s) of Ni(OH)_2_ and Pt-Ni(OH)_2_ with/without EtOH. (e) *In situ* Raman spectrum of Ni(OH)_2_ during the OER and AOR. (f) *In situ* Raman spectrum of Pt-Ni(OH)_2_ during the OER and AOR. (g) Nyquist plots at 1.35 V vs. RHE. (h) Bode phase plots at 1.55 V vs. RHE. (i) The product distribution of the reaction over NiO, Ni(OH)_2_ and Pt-Ni(OH)_2_.

Cation-induced aldehyde aggregation and increased local CH_3_CHO concentration around PhCHO improves coupling kinetics. Based on this, we hypothesized that a catalyst with faster alcohol oxidation could enhance PhCHO–CH_3_CHO interactions by generating more CH_3_CHO intermediates, further promoting aggregation. Thus, we focused on developing the anode catalyst. With this idea in mind, we synthesized a platinum-loaded Ni(OH)_2_ catalyst (Pt-Ni(OH)_2_) via a hydrothermal method using Ni(OH)_2_ as the precursors, which is a good electrocatalyst for the alcohol oxidation reaction [37]. Figure 3a shows that the diffraction peaks of Pt/Ni(OH)_2_ could be indexed to cubic-phased Ni(OH)_2_ (JCPDS 14-0117) and cubic-phased Pt (JCPDS 04-0802), respectively, indicating that Pt-Ni(OH)_2_ was successfully synthesized. XPS was used to probe the surface chemical composition of the materials of the prepared samples. As shown in Fig. 3b, the Pt-Ni(OH)_2_ sample exhibits prominent characteristic peaks at binding energies of 71.5 and 74.7 eV, corresponding to Pt 4*f*_7/2_ and 4*f*_5/2_ of Pt^0^, respectively. The electronic interaction between Pt and Ni(OH)_2_ is proved by the positive binding energy shift of Pt 4f compared to Pt/C. It is also discerned by Ni 2p XPS spectra that there is no significant change in the chemical state of Ni (II) after Pt loading on Ni(OH)_2_ surfaces (Fig. 3b and c). The Pt content in Pt-Ni(OH)_2_ is ∼11.6 wt%, which is quantitatively measured with an inductively coupled plasma mass spectrometry (ICP-MS) technique.

EtOH oxidation performance was evaluated on Pt-Ni(OH)_2_ and Ni(OH)_2_. Pt-Ni(OH)_2_ showed an advanced oxidation peak and larger catalytic current for EtOH oxidation versus Ni(OH)_2_, indicating faster EtOH oxidation kinetics (Fig. 3d). The evolution of surface species on the Ni(OH)_2_ electrode was carried out to analyze the active sites of the reaction. *In situ* Raman spectroscopy was used to monitor the bending and stretching vibrations of Ni^3+^–O at 473 and 553 cm^−1^. For Ni(OH)_2_, Ni^3+^–O species are accumulated after 1.40 V (no EtOH) and 1.55 V (100 mM EtOH), respectively. For the Pt-Ni(OH)_2_, Ni^3+^–O species are accumulated after 1.35 V when no EtOH is present, meaning that Pt loading can facilitate the generation of Ni^3+^–O. Furthermore, Ni^3+^–O species cannot be accumulated within 1.55 V when 100 mM EtOH is present, indicating enhanced reaction dynamics due to the induction effects of Pt (Fig. 3e and f). *In situ* electrochemical impedance spectroscopy (EIS) was further adopted to probe the interface behavior under different potentials. As shown in Fig. 3g and Fig. S24, the presence of Pt promoted the electron transfer of Pt-Ni(OH)_2_ with smaller charge transfer resistance (*R*_ct_) at a relatively lower voltage, verifying the positive effect of Pt loading. The phase angle value of the Pt-Ni(OH)_2_ material in the low-frequency region is significantly lower than that of Ni(OH)_2_, which means that the reaction kinetics of the composite are faster (Fig. 2h and Fig. S25).

Chronoamperometry experiments were conducted for the electrochemical co-oxidation coupling reaction, demonstrating that Pt-Ni(OH)_2_ catalysts can achieve the same current density as unmodified Ni(OH)_2_ with lower energy input (Fig. S26). Using Pt-Ni(OH)_2_ as the anodic catalyst resulted in increased conversion of the alcohol substrates as well as higher selectivity towards the desired product CAL (Fig. 3i and Fig. S27). In contrast, nickel oxide (NiO) catalysts with poor alcohol oxidation ability gave decreased yields and faradaic efficiencies. Notably, a cyclic hydrodimer (CHD) byproduct was observed during the reaction, with the CHD selectivity increasing concurrently with CAL. In particular, the Pt-Ni(OH)_2_ catalyst with superior performance produced a 30% yield of CHD. Furthermore, a control experiment was conducted in the divided cell using CAL as the substrate, and the resulting gas chromatography mass spectrometry (GC-MS) spectra are presented in Fig. S28 after the reaction. The spectra reveal the presence of CHD products, indicating CHD is likely formed through reduction of CAL.

**Figure 4.**
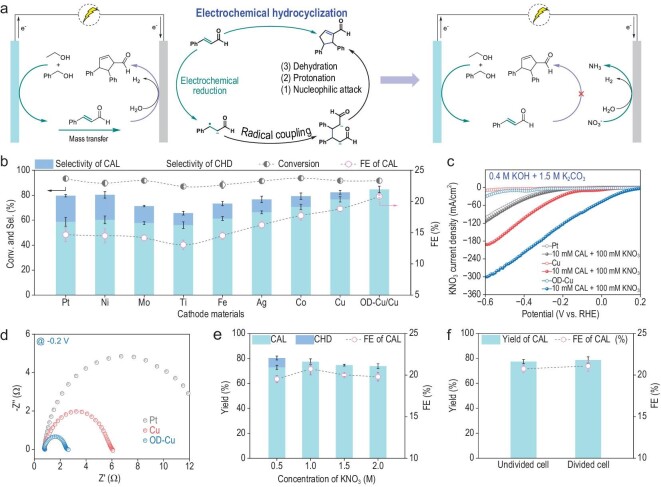
Cathodic replacing reaction improves coupling selectivity. (a) The mechanism and inhibition strategy of the CHD. (b) The product distributions with different cathodes. (c) LSV curves of the different cathodes with/without CAL and KNO_3_. (d) Nyquist plots at −0.2 V vs. RHE. (e) The effect of KNO_3_ concentration on the reaction. (f) The yield and FE of the reaction in an undivided cell and divided cell, respectively.

To elucidate the mechanism of CHD generation, the electrochemical process at the cathode was investigated. LSV of the hydrogen evolution reaction (HER) was performed using a platinum mesh cathode in mixed 0.4 M KOH and varying concentrations of K_2_CO_3_ (Fig. S29). Increasing the K_2_CO_3_ concentration resulted in decreased HER current density. This can be attributed to the formation of hydration shells around K^+^ ions by water molecules, reducing availability of protons required for HER. We hypothesize that the production of CHD stems from the increased salt concentration lowering the cathode proton source, such that the hydrogen evolution current cannot match the anodic alcohol oxidation current. Under these conditions, CAL produced at the anode transfers to the cathode and undergoes reductive dimerization to generate CHD (Fig. 4a). This provides the required cathodic current to balance the anodic current, but decreases the yield of the desired CAL product.

Inhibiting the reduction of CAL at the cathode is critical for obtaining high selectivity and yield for the electrocatalytic coupling reaction. An effective solution is to introduce a thermodynamic, more favorable alternative reaction at the cathode, which is more readily reducible than CAL and even than HER; this would avoid the reduction of CAL at the cathode. As a result, we focus our attention on replacing the HER at the cathode. The electrocatalytic nitrate reduction reaction (NO_3_^−^RR) has made significant progress recently, both fundamentally and for practical applications in NH_3_ production. Theoretically, the potential for NO_3_^−^RR is +0.69 V vs. the reversible hydrogen electrode (RHE) under alkaline conditions [38]. This potential is much more positive than that for the HER. Given its more favorable thermodynamics, implementing NO_3_^−^RR at the cathode can effectively replace HER and avoid the undesired reduction of organic compounds like CAL. Compared with the two-electron alcohol oxidation reaction, NO_3_^−^RR is an eight-electron process. This means the production rate of aldehydes is much faster than that of ammonia. Controlled experiments demonstrate that both PhCHO and CAL can achieve higher raw material recovery across a range of ammonia concentrations from 0 to 200 mM (Fig. S30). Using PhCH_2_OH and NH_3_ as substrates, PhCOOH was the primary product after electrolysis. Only small amounts of C−N coupled products formed even at 200 mM ammonia (Fig. S31). The highly positive potential of NO_3_^−^RR render it an ideal cathodic reaction to pair with the electro-oxidation of alcohols at the anode.

Keeping the K^+^ ion concentration at 3.4 M, the effects of nine cathode catalysts (Pt, Ni, Mo, Ag, Ti, Fe, Co, Cu and oxide-derived-Cu (OD-Cu) foil) on the reaction were evaluated in the electrolyte of 0.4 M KOH, 1.0 M K_2_CO_3_ and 1.0 M KNO_3_ (Fig. 4b). Of these, eight metal foils were purchased commercially, and OD-Cu was synthesized by a wet chemical oxidation method, followed by electrochemical reduction to increase the electrochemical surface area. With nitrate addition, CAL selectivity remained largely unchanged with a Pt cathode. Ni, Mo and Ti cathodes resulted in lower CAL selectivity and faradaic efficiency. In contrast, Fe, Ag, Co and Cu increased CAL selectivity and decreased CHD. Excitingly, the OD-Cu cathode achieved 91% conversion with 85% CAL selectivity and no detected CHD.

The electrochemical activity for NO_3_^−^RR was investigated on different cathode electrocatalysts as shown in Fig. 4c and Fig. S32. It is observed that OD-Cu exhibited excellent NO_3_^−^RR activity compared to the other catalysts tested. This explains the high CAL selectivity obtained with the OD-Cu cathode, since the use of OD-Cu as a catalyst completely inhibited the undesired cathodic reduction of CAL. Next, charge transfer characteristics were studied by potentiostatic EIS in 0.4 M KOH and 1.0 M K_2_CO_3_ with 1.0 M KNO_3_ electrolyte at −0.2 V vs. RHE (Fig. 4d). OD-Cu showed lower charge transfer resistance than Pt and Cu cathodes, indicating faster charge transfer kinetics with OD-Cu. This agrees with and helps explain the higher NO_3_^−^RR activity of OD-Cu observed. As a result, the high NO_3_^−^RR activity and kinetics of OD-Cu enabled inhibition of CAL reduction at the cathode and improved selectivity.

The effect of nitrate concentration was evaluated (Fig. 4e). With 0.5 M KNO_3_, 7% CHD yield was detected, decreasing CAL yield. However, CAL yield also decreased at KNO_3_ concentrations above 1.0 M. This may be due to anodic oxidation of nitrogen-containing intermediates (e.g. NO_2_^−^, NH_2_OH and NH_3_) formed during nitrate reduction, competing with alcohol oxidation. Using a divided cell resulted in 79% CAL yield (Fig. 4f). Compared with a divided cell, pairing with nitrate reduction maintains almost unchanged conversion and selectivity without expensive membranes, making it a promising HER alternative in undivided electrolytic cells. Moreover, this strategy only required a cell voltage of 1.63 V, lower than Ni(OH)_2_|Pt (2.22 V), Pt-Ni(OH)_2_|Pt (2.03 V) and Pt-Ni(OH)_2_|OD-Cu (1.71 V) (Fig. S33). The reaction temperature effect was also evaluated (Fig. S34). Higher temperatures decreased CAL selectivity and increased phenylacetic acid, likely due to accelerated aldehyde hydration.

**Figure 5.**
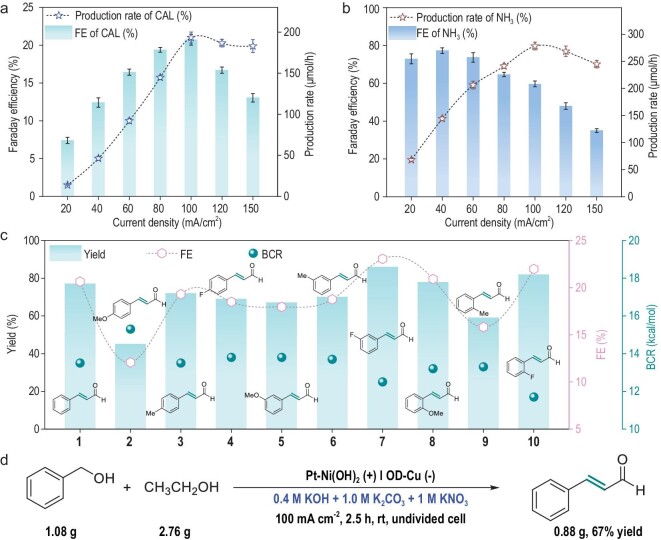
Reaction scalability and scope of substrate. (a) The yield and production rate of CAL at different current densities. (b) The Faraday efficiency and production rate of NH_3_ at different current densities. (c) Scope of the alcohol oxidation coupling reaction. (d) Scale-up experiment. Reaction conditions: substrate (100 mM aromatic alcohol; 600 mM EtOH), 5 mL 0.4 M KOH + 1.0 M K_2_CO_3_ + 1.0 M KNO_3_ solution, Pt-Ni(OH)_2_/NF anode, OD-Cu foil cathode, 100 mA/cm^2^, room temperature, undivided cell.

We investigated the yield and production rate of CAL at different current densities. As shown in Fig. 5a, the production rate of CAL reached a maximum of 193 μmol/h at 100 mA/cm^2^. Deviating from this optimal current density decreased both the yield and production rate. Furthermore, the faradaic efficiency (FE) and production rate of NH_3_ were detected by ultraviolet and visible spectrophotometry (UV-Vis) at different currents (Fig. 5b and Fig. S36). At 100 mA/cm^2^, 60% FE of NH_3_ was achieved, corresponding to 278 μmol/h production. This optimized paired electrolysis system demonstrated good stability over six cycles, with no decrease in yield and FE (Figs S37 and S38). On the other hand, after the reaction, Pt-Ni(OH)_2_ maintained its original morphology, crystalline phase and surface chemical valence states, indicating decent catalyst stability (Figs S39–S42).

The generality of this protocol was investigated using various substituted aromatic alcohols. Alcohols with electron-rich and electron-deficient substituents (**1–10**, Fig. 5c) successfully produced the corresponding CAL coupling products. Notably, ortho- and meta-fluorine substituents gave 2-F (**10**, 82% yield) and 3-F (**7**, 86% yield) CALs, respectively. Methoxy-substituent at the para-position and meta-position results in 45% yield of the 4-OMe CAL (**2**) and 67% yield of 4-OMe CAL (**e**), respectively. Combining experimental and DFT calculated BDFEs for the alcohols and condensation reaction barriers (BCR), alcohols with lower BDFE and especially lower BCR gave higher yields and FE (e.g. **7** and **10**). In contrast, higher alcohol BDFE and BCR resulted in lower yields (**4** and **6**) (Figs S43 and S44). Additionally, a lower alcohol BDFE but higher aldehyde BCR also caused lower yield (**2**), likely due to slower condensation kinetics and buildup of unreacted aldehyde occupying active sites. Importantly, gram-scale synthesis of CAL with 67% yield was achieved under identical conditions in a 100-mL beaker, demonstrating scalability and robustness (Fig. 5d).

## CONCLUSION

In summary, efficient electrocatalytic co-oxidative coupling of ethanol and benzyl alcohol to CAL was achieved through three key strategies: (i) suppressing alcohol over-oxidation to acids using the salt-out effect; (ii) accelerating cross-coupling of intermediates with a Pt-Ni(OH)_2_ catalyst that has faster kinetics than Ni(OH)_2_; (iii) replacing HER with nitrate reduction to provide more favorable thermodynamics, higher value ammonia, reduced energy use and improved selectivity by inhibiting hydrogenation. Under optimal conditions, 91% conversion and 85% selectivity to CAL were obtained along with 278 μmol/h NH_3_ productive rate in a single electrolysis cell. The substrate tolerance and scaled-up experiments demonstrate practicability. This work provides a universal design principle for electrocatalytic alcohol oxidation to value-added carbon products.

## METHODS

### Synthesis of Ni(OH)_2_

Ni(NO_3_)_2_·6H_2_O (12 mmol) was dissolved in 40 mL deionized water and stirred for 15 min. The 0.6 M NaOH aqueous solution was then added until the final pH of the mixed suspension was >13. After stirring for 30 min, the Ni(OH)_2_ suspension was transferred to a 100-mL Teflon-lined stainless-steel autoclave and maintained at 160ºC for 6 h. The autoclave was cooled down naturally to room temperature. The Ni(OH)_2_ was washed three times with deionized water and anhydrous ethanol and dried at 60ºC for 10 h. Finally, the Ni(OH)_2_ was obtained.

### Synthesis of Ni(OH)_2_/NF

Ni(NO_3_)_2_ (10 mmol) and NH_4_NO_3_ (5 mmol) were dissolved with 32 mL deionized water and stirred for 15 min. 18 mL ammonia (28 wt%) was then added with vigorous stirring. After stirring for 15 min, the mixed solution was poured into a culture dish. The culture dish with the solution was preheated at 90°C for 2 h. Then the clean nickel foam (NF) was placed in the mixed solution and maintained at 90°C for 12 h. The Ni(OH)_2_/NF was washed three times with deionized water and anhydrous ethanol and dried at 60°C for 10 h. Finally, the Ni(OH)_2_/NF was obtained.

### Synthesis of Pt-Ni(OH)_2_

Ni(OH)_2_ (50 mg) was dispersed in 50 mL ethylene glycol and sonicated for 30 minutes. 1.66 mL H_2_PtCl_6_·6H_2_O solution (20 mg/mL) was added to achieve a 20 wt% Pt loading. The mixture was stirred for additional 30 minutes. The solution was then transferred to a 100-mL polytetrafluoroethylene (PTFE)-lined autoclave and reacted at 120°C for 4 h. After cooling to room temperature, the solid was collected by filtration and dried under vacuum overnight at 60°C.

### Synthesis of oxide-derived-Cu

OD-Cu/Cu foil was prepared by a wet chemical oxidation method, followed by electrochemical reduction. Specifically, one piece of Cu foil was successively washed for 10 min in 2 M HCl solution, ethanol and water, respectively. Thereafter, the Cu foil was immersed in an aqueous solution (2.67 M NaOH and 0.13 M (NH_4_)_2_S_2_O_8_) for 30 min at room temperature in order to grow Cu(OH)_2_ nanorods on the surface. The resulting Cu(OH)_2_/Cu foil was washed by water three times and dried at 60°C for 12 h. Before use, the Cu(OH)_2_/Cu foil was electrochemically reduced into OD-Cu/Cu foil at −3 V for 30 min in 1 M KOH solution.

## Supplementary Material

nwae134_Supplemental_File

## References

[1] Mo Y, Lu Z, Rughoobur G et al. Microfluidic electrochemistry for single-electron transfer redox-neutral reactions. Science 2020; 368: 1352–7. 10.1126/science.aba3823 32554592

[2] Leow W, Lum Y, Ozden A et al. Chloride-mediated selective electrosynthesis of ethylene and ropylene oxides at high current density. Science 2020; 368: 1228–33. 10.1126/science.aaz8459 32527828

[3] Yan M, Kawamata Y, Baran P. Synthetic organic electrochemical methods since 2000: on the verge of a renaissance. Chem Rev 2017; 117: 13230–319. 10.1021/acs.chemrev.7b00397 28991454 PMC5786875

[4] Wang H, Gao X, Lv Z et al. Recent advances in oxidative R^1^-H/R^2^-H cross-coupling with hydrogen evolution via photo-/electrochemistry. Chem Rev 2019; 119: 6769–87. 10.1021/acs.chemrev.9b00045 31074264

[5] Kingston C, Palkowitz M, Takahira Y et al. A survival guide for the “electro-curious”. Acc Chem Res 2020; 53: 72–8. 10.1021/acs.accounts.9b00539 31823612 PMC6996934

[6] Wang F, Stahl S. Electrochemical oxidation of organic molecules at lower overpotential: accessing broader functional group compatibility with electron-proton transfer mediators. Acc Chem Res 2020; 53: 561–74. 10.1021/acs.accounts.9b00544 32049487 PMC7295176

[7] Horn E, Rosen B, Chen Y et al. Scalable and sustainable electrochemical allylic C–H oxidation. Nature 2016; 533: 77–8. 10.1038/nature17431 27096371 PMC4860034

[8] Xiang J, Shang M, Kawamata Y et al. Hindered dialkyl ether synthesis with electrogenerated carbocations. Nature 2019; 573: 398–402. 10.1038/s41586-019-1539-y 31501569 PMC6996793

[9] Gnaim S, Takahira Y, Wilke H et al. Electrochemically driven desaturation of carbonyl compounds. Nat Chem 2021; 13: 367–72. 10.1038/s41557-021-00640-2 33758368 PMC8049972

[10] Liang Y, Shi S, Jin R et al. Electrochemically induced nickel catalysis for oxygenation reactions with water. Nat Catal 2021; 4: 116–23. 10.1038/s41929-020-00559-w

[11] Selt M, Franke R, Waldvogel S. Supporting-electrolyte-free and scalable flow process for the electrochemical synthesis of 3,3′,5,5′-tetramethyl-2,2′-biphenol. Org Proc Res & Dev 2020; 24: 2347–55.

[12] Stephen H, Schotten C, Nicholls T et al. A versatile electrochemical batch reactor for synthetic organic and inorganic transformations and analytical electrochemistry. Org Proc Res & Dev 2020; 24: 1084–9.

[13] Xia R, Tian D, Kattel S et al. Electrochemical reduction of acetonitrile to ethylamine. Nat Commun 2021; 12: 1949. 10.1038/s41467-021-22291-0 33782400 PMC8007591

[14] He Z, Hwang J, Gong Z et al. Promoting biomass electrooxidation via modulating proton and oxygen anion deintercalation in hydroxide. Nat Commun 2022; 13: 3777. 10.1038/s41467-022-31484-0 35773257 PMC9246976

[15] Yang G, Jiao Y, Yan H et al. Unraveling the mechanism for paired electrocatalysis of organics with water as a feedstock. Nat Commun 2022; 13: 3125. 10.1038/s41467-022-30495-1 35668075 PMC9170728

[16] Huang Y, Chong X, Liu C et al. Boosting hydrogen production by anodic oxidation of primary amines over a NiSe nanorod electrode. Angew Chem Int Ed 2018; 57: 13163–6. 10.1002/anie.201807717 30118157

[17] Ge R, Wang Y, Li Z et al. Selective electrooxidation of biomass-derived alcohols to aldehydes in a neutral medium: promoted water dissociation over a nickel-oxide-supported ruthenium single-atom catalyst. Angew Chem Int Ed 2022; 61: e20220021. 10.1002/anie.202200211 35170172

[18] Chen W, Xie C, Wang Y et al. Activity origins and design principles of nickel-based catalysts for nucleophile electrooxidation. Chem 2020; 6: 2974–93. 10.1016/j.chempr.2020.07.022

[19] Meng N, Shao J, Li H et al. Electrosynthesis of formamide from methanol and ammonia under ambient conditions. Nat Comm 2022; 13: 5452. 10.1038/s41467-022-33232-w PMC9481544 36114196

[20] Shao J, Meng N, Wang Y et al. Scalable electrosynthesis of formamide through C–N coupling at the industrially relevant current density of 120 mA cm^−2^. Angew Chem Int Ed 2022; 61: e202213009. 10.1002/anie.202213009 36106683

[21] Badalyan A, Stahl S. Cooperative electrocatalytic alcohol oxidation with electron-proton-transfer mediators. Nature 2016; 535: 406–10. 10.1038/nature18008 27350245

[22] Wang D, Wang P, Wan S et al. Direct electrochemical oxidation of alcohols with hydrogen evolution in continuous-flow reactor. Nat Commun 2019; 10: 2796. 10.1038/s41467-019-10928-0 31243290 PMC6594969

[23] Bender M, Yuan X, Choi K. Alcohol oxidation as alternative anode reactions paired with (photo)electrochemical fuel production reactions. Nat Commun 2020; 11: 4594. 10.1038/s41467-020-18461-1 32929086 PMC7490346

[24] Liu F, Gao X, Shi R et al. Concerted and selective electrooxidation of polyethylene-terephthalate-derived alcohol to glycolic acid at an industry-level current density over a Pd-Ni(OH)_2_ catalyst. Angew Chem Int Ed 2023; 62: e202300094. 10.1002/anie.202300094 36656087

[25] Zhang N, Zou Y, Tao L et al. Electrochemical oxidation of 5-hydroxymethylfurfural on nickel nitride/carbon nanosheets: reaction pathway determined by in situ sum frequency generation vibrational spectroscopy. Angew Chem Int Ed 2019; 58: 15895–903. 10.1002/anie.201908722 31452306

[26] Wu J, Kong Z, Li Y et al. Unveiling the adsorption behavior and redox properties of PtNi nanowire for biomass-derived molecules electrooxidation. ACS Nano 2022; 16: 21518–26. 10.1021/acsnano.2c10327 36475597

[27] Lu Y, Dong C, Huang Y et al. Hierarchically nanostructured NiO-Co_3_O_4_ with rich interface defects for the electro-oxidation of 5-hydroxymethylfurfural. Sci China Chem 2020; 63: 980–98. 10.1007/s11426-020-9749-8

[28] Lu Y, Liu T, Huang Y et al. Integrated catalytic sites for highly efficient electrochemical oxidation of the aldehyde and hydroxyl groups in 5-hydroxymethylfurfural. ACS Catal 2022; 12: 4242–51. 10.1021/acscatal.2c00174

[29] Chen W, Shi J, Xie C et al. Unraveling the electrophilic oxygen-mediated mechanism for alcohol electrooxidation on NiO. Natl Sci Rev 2023; 10: nwad099. 10.1093/nsr/nwad099 37287808 PMC10243987

[30] Chen W, Wang Y, Wu B et al. Activated Ni–OH bonds in a catalyst facilitates the nucleophile oxidation reaction. Adv Mater 2022; 34: 2105320. 10.1002/adma.202105320 35472674

[31] Palomo C, Oiarbide M, García J. Current progress in the asymmetric aldol addition reaction. Chem Soc Rev 2004; 33: 65–75. 10.1039/B202901D 14767502

[32] Enache D, Edwards J, Landon P et al. Solvent-free oxidation of primary alcohols to aldehydes using Au-Pd/TiO_2_ catalysts. Science 2006; 311: 362–5. 10.1126/science.1120560 16424335

[33] Lu Y, Li Y, Zhou B et al. Anodic electrosynthesis of amide from alcohol and ammonia. CCS Chemistry 2024; 6: 125–36. 10.31635/ccschem.023.202302727

[34] Min S, Kim N, Seo S et al. Recyclable palladium catalyst for highly selective α alkylation of ketones with alcohols. Angew Chem Int Ed 2005; 44: 6913–5. 10.1002/anie.200502422 16206316

[35] Baruah M, Sharma M, Das B et al. Boosting multiple photo-assisted and temperature controlled reactions with a single redox-switchable catalyst: solvents as internal substrates and reducing agent. J Catal 2020; 388: 104–21. 10.1016/j.jcat.2020.04.026

[36] Xu L, Huang Z, Yang M et al. Salting-out aldehyde from the electrooxidation of alcohols with 100 % selectivity. Angew Chem Int Ed 2022; 61: e202210123. 10.1002/anie.202210123 36073150

[37] Zhou B, Li Y, Zou Y et al. Platinum modulates redox properties and 5-hydroxymethylfurfural adsorption kinetics of Ni(OH)_2_ for biomass upgrading. Angew Chem Int Ed 2021; 60: 22908–14. 10.1002/anie.202109211 34405508

[38] Wang Y, Xu A, Wang Z et al. Enhanced nitrate-to-ammonia activity on copper–nickel alloys via tuning of intermediate adsorption. J Am Chem Soc 2020; 142: 5702–8. 10.1021/jacs.9b13347 32118414

