# Supplementary material for: Combining anodic alcohol oxidative coupling for C–C bond formation with cathodic ammonia production
Source: Natl Sci Rev. 2024 Apr 4;11(5):nwae134. doi: 10.1093/nsr/nwae134 (PMC11092398; doi:10.1093/nsr/nwae134)
Supplement: nwae134_Supplemental_File [file nwae134_supplemental_file.docx]

Supporting Information

Combining anodic alcohol oxidative coupling for C-C bond formation with cathodic ammonia production

Leitao Xu^‡^, Wei Chen^‡^, Cairong Wang^‡^, Wenjie Wu, Yelin Yao, Zhifeng Huang, Jingcheng Wu, Ming Yang, Yandong Wu, Dianke Xie, Yuqin Zou and Shuangyin Wang*

State Key Laboratory of Chemo/Bio-Sensing and Chemometrics, College of Chemistry and Chemical Engineering, Hunan University, Changsha, Hunan, 410082 (P. R. China)

^‡^These authors contributed equally to this work.

*Corresponding author.

E-mail address: [shuangyinwang@hnu.edu.cn](mailto:shuangyinwang@hnu.edu.cn;)

**Method**

**Characterizations.**

The crystal structure of the electrocatalyst was identified by X-ray diffraction (XRD, Bruker D8 Advance diffractometer, Cu Kα1). The comprehensive morphology structure of the electrocatalyst was characterized by the scanning electron microscope (SEM, Hitachi, S-4800). X-ray photoelectron spectroscopy (XPS) measurements and analysis were recorded on AXIS Supra (Axis Supra, Kratos, England). All XPS spectra were adjusted by C 1s (284.8 eV). ^1^H and ^13^C NMR spectra were obtained with Bruker 400 MHz at 20 °C with CDCl_3_, and DMSO-d6 as solvent. Chemical shifts (ppm) are given relative to solvent: references for CDCl_3_ were 7.26 ppm (^1^H NMR) and 77.16 ppm (^13^C NMR); references for DMSO-d6 were 2.50 ppm (^1^H NMR) and 39.52 ppm (^13^C NMR); The abbreviations used for explaining the multiplicities were as follows: s = singlet, d = doublet, t = triplet, q = quartet, m = multiplet.

**Electrochemical measurements.**

All electrochemical measurements were performed by an electrochemical workstation (CHI 760D, CH Instruments Ins, Shanghai). The glassy carbon electrode was carried out to explore the alcohol oxidation mechanism and reaction kinetics over the Ni(OH)_2_ electrode. Firstly, 4 mg electrocatalyst was dispersed in a mixed solution including 475 μL deionized water and 475 μL 2-propanol with ultrasonic dispersion for 20 min. Secondly, 50 µL Nafion solution (5 wt%) was injected into the homogeneous suspension with ultrasonic dispersion for 20 min. Then 10 μL ink was dropped onto the surface of the glass carbon electrode (diameter: 5 mm). Ultimately, on the glassy carbon electrode, a thin-film electrode (0.2 mg cm^-2^ electrocatalyst) was prepared and employed in the electrochemical measurement. It is worth noting that, for Raman spectra, Nafion was not added during thin film electrode fabrication to avoid the influence of polymer. All electrochemical measures were carried out in the three-electrode system with the thermostatic water bath (25°C). The carbon rod and Hg/HgO electrode were adopted as counter and reference electrodes, respectively. All electrochemical data was converted to the reversible hydrogen electrode (RHE). The reversible hydrogen electrode (RHE) was determined with a platinum gauze electrode as a working electrode in the hydrogen-saturated electrolyte solution.

**In Situ Raman Spectrum Tests.**

In situ Raman spectrum was carried out on the confocal Raman microscope (Alpha300R, WETEC, Germany) under different potentials by CHI 760D electrochemical workstation. The electrolytic cell for in situ Raman spectrum comprises a Teflon shell, quartz glass plate, and glassy carbon electrode. For powdered catalysts, the catalysts (4 mg) were mixed with deionized water (1 mL) with ultrasonic dispersion for 20 min. 6.4 mL of the catalyst suspension (0.2 mg cm^2^) was dropped on a glassy carbon electrode (4 mm in diameter). All of the electrochemical tests were carried out with the three-electrode configuration. During the measurements, the reference electrode was SCE. The counter electrode was a platinum wire for HER. The SCE reference electrode was corrected by testing the RHE potential with a platinum gauze electrode in a 1 M KOH hydrogen-saturated solution. For the OER system, the electrolyte is 1 M KOH. For the alcohol oxidation reaction system, the electrolyte is 1 M KOH with 100 mM ethanol.

**General experiment procedure**

(a) Electrocatalytic coupling synthesis of 2a-2k: In an undivided round-bottomed reactor, 5 mL of the mix of 0.4 M KOH, 1.0 M K_2_CO_3_, and 1.0 M KNO_3_ as the electrolyte in solution. Then added 100 mM PhCH_2_OH and 600 mM EtOH into the reactor. The Pt-Ni(OH)_2_/NF was used as the working electrode with an exposed surface area of (1.0 cm × 1.0 cm) and a OD-Cu foil (1.0 cm × 1.0 cm) as the cathode. The electrolysis was carried out at room temperature using a constant current of 100 mA. After 2-3 hours of reaction, the electrolyte was neutralized by 1 M HCl. The products were extracted by dichloromethane (DCM) and purified by flash column chromatography. GC yields were reported.

(b) Electrocatalytic coupling synthesis of CAL on gram scale: In a 150 mL beaker, 100 mL mix of 0.4 M KOH, 1.0 M K_2_CO_3_, and 1.0 M KNO_3_ as the electrolyte in solution. Then added 100 mM PhCH_2_OH and 600 mM EtOH into the reactor. The Pt-Ni(OH)_2_/NF was used as the working electrode with an exposed surface area of (4.0 cm × 5.0 cm) and a OD-Cu foil (4.0 cm × 5.0 cm) as the cathode. The electrolysis was carried out at room temperature using a constant current of 1.6 A. After 3 hours of reaction, the products were extracted by dichloromethane (DCM) and the residue was chromatographed through silica gel eluting with petroleum ether/ethyl acetate (40:1) to give the desired product. Isolated yields were reported.

**Products analysis**

The aromatic products were analyzed qualitatively by gas chromatography-mass spectrometry (GC-MS) equipped with an HP-5MS capillary column. The oven temperature was increased to 50°C from room temperature for 1 min, and then ramp to 320°C with 30°C /min and hold for 5 min. Helium was the carrier gas with a split ratio of 9:1. The conversion of EtOH and selectivity of acetic acid were analyzed qualitatively by ^1^H NMR spectra. The conversion and selectivity were analyzed by the following equations:

$Conversion\text{ }(\%)\text{ }=\text{ }\frac{Initial\text{ }mole\text{ }of\text{ }PhCH_{2}OH-Final\text{ }mole\text{ }of\text{ }PhCH_{2}OH}{Initial\text{ }mole\text{ }of\text{ }PhCH_{2}OH\text{ }}\text{ }\times\text{ }100\%$ $Selectivity\text{ }(\%)\text{ }=\text{ }\frac{mole\text{ }of\text{ }certain\text{ }product\text{ }}{mole\text{ }of\text{ }comsumed\text{ }substrate}\text{ }\times\text{ }100\%$ $Yield\text{ }(\%)\text{ }=\text{ }Conversion\text{ }\times\text{ }Selectivity\text{ }\times\text{ }100\%$

**Determination of NH_3_**

The yield of the NH_3_ was determined by a spectrophotometry measurement with the indophenol blue method. For indophenol blue method, the concentration-absorbance curves were calibrated using a standard NH_3_ solution with a series of concentrations. The fitting curve (y = 0.23831x + 0.02455, R^2^ = 0.9998) shows good linear relation of absorbance value with NH_3_ concentration.

**Computational methods**

**(a) Reaction mechanism, hydration energy barrier, and BDFE calculation**

All structures were optimized in the gas phase at M06-2X^1^/6-31+G (d,p)^2^ level. Harmonic frequency analysis calculations were subsequently performed to verify the optimized geometries to be minima (no imaginary frequency) or transition states (TSs, with unique one imaginary frequency) to provide thermal contributions of free energies at 298.15 K. The transition states were checked through intrinsic reaction coordinate (IRC)calculations. Improved energies were computed at M06-2X/ ma-def2-TZVP^3^ single-point calculations including solvation effects with the SMD^4^ continuum solvation model. All calculations were carried out using Gaussian 16 program^5^.

**(b) MD simulations**

MD simulations were carried out with the open-source software Gromacs (version. 2018). During model construction, 10 benzyl alcohol (100 mM) and 60 ethanol molecules (600 mM) or 10 benzaldehyde and 60 acetaldehyde molecules were randomly dispersed in a K_2_CO_3_ solution (0-4 M), which consisted of 0-800 K^+^ and 0-400 CO_3_^2-^ ions in 5500 water molecules. For water, organic molecules, and K_2_CO_3_, the SPC/E model^6^, TraPPE rigid model^7^, and parameters from Chowdhuri et al^8^. were employed, respectively. To generate the partial charges, the molecular geometry was optimized at the M06-2X/6-311G(d,p) level in Gaussian 16 software, and then analyzed by the Multiwfn program^9^ to gain RESP charges for use in MD simulations. vdW interactions were described by the Lennard Jones (LJ) potential^10^, which was truncated at 1.0 nm. The LJ interaction parameters between unlike atom pairs were generated by the standard Lorentz-Berthelot combination rule.^11-12^ Electrostatic interactions were calculated with the particle mesh Ewald method, with the short-range part truncated at 1.2 nm and the long-range part calculated in the reciprocal space with a Fourier spacing of 0.12 nm. The equations of motion were integrated by the leapfrog algorithm with a time step of 1 fs. The intermolecular interactions are expressed as the sum of the vdW interactions described by the LJ potential and the Coulombic interactions ^13-14^:

$$U_{ij}=4\times\varepsilon_{ij}\times\left[\left(\sigma_{ij}/r_{ij}\right)^{12}-\left(\sigma_{ij}/r_{ij}\right)^{6}\right]+\left(q_{i}\times q_{j}/r_{ij}\right)$$

where r_ij_ is the distance between particles *i* and *j*, σ and ε are the size parameter and energy parameter, respectively, and *q*_i_ is the charge of the *i*th atom (or ion). The potential parameters for unlike site pairs are expressed via the LB mixing rules:

$$\sigma_{ij}=\left(\sigma_{i}+\sigma_{j}\right)/2$$

$$\varepsilon_{ij}=\left(\varepsilon_{i}\times\varepsilon_{j}\right)^{1/2}$$

Initial configurations were energy minimized by the steepest descent algorithm, followed by 200 ps equilibration at 298 K and 1 bar. Then, 20 ns MD runs were performed under the NPT ensemble (298 K, 1 bar). The system temperature and pressure were controlled with a Nose-Hoover thermostat and Parrinello-Rahman barostat. Three-dimensional periodic boundary conditions were applied throughout the simulations. To evaluate the H_2_O accumulation around the organic molecules, the integrated RDFs were calculated as follows:

$$n_{ji}\left(r\right)=4\times\pi\times\rho_{j}\times\int_{0}^{r} g_{ji}\left(r\right)r^{2}dr$$

where *g*_ji_ is the RDF of *j* about *i*, *ρ*_j_ is the bulk number density of *j*, and *r* is the intercept radius.

**Supplementary Figures.**

**Supplementary Fig. 1.** The mechanism of alcohol oxidation reaction over the Ni(OH)_2_ electrode

**Supplementary Fig. 2.** XRD pattern for Ni(OH)_2_.


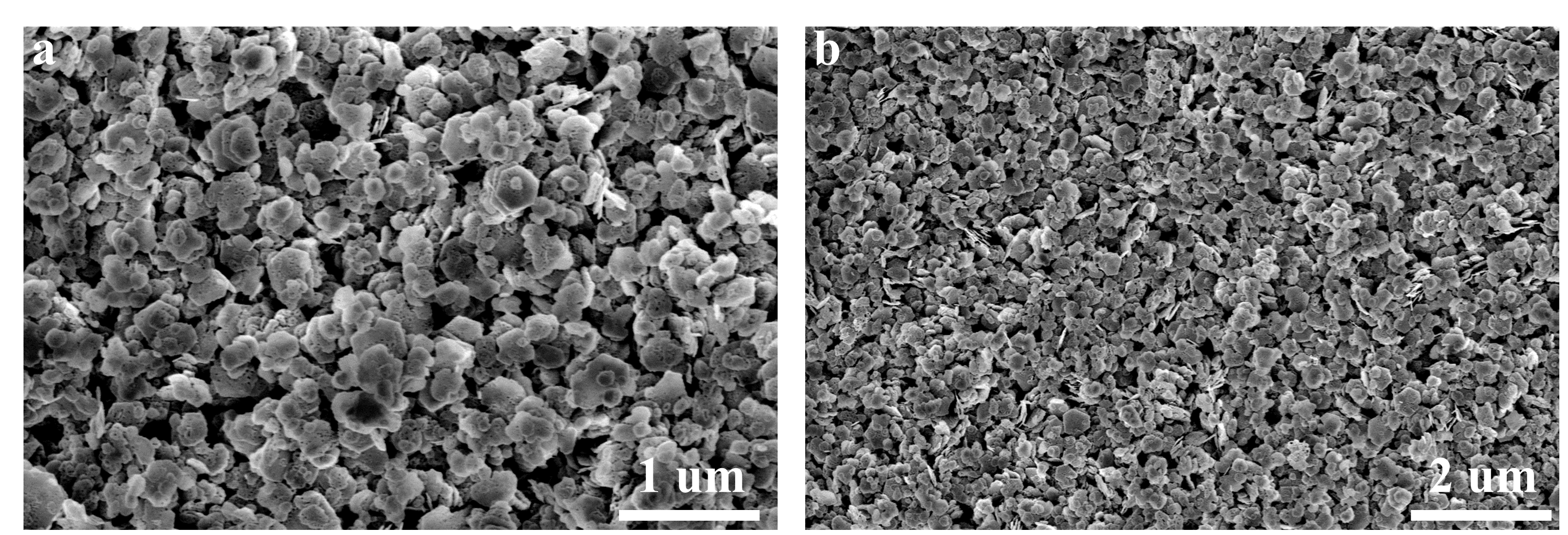


**Supplementary Fig. 3.** SEM images of (a, b, and c) Ni(OH)_2_.

**Supplementary Fig. 4.** XPS spectra of Ni(OH)_2_.

**Supplementary Fig. 5.** LSV (10 mV/s) curves of Ni(OH)_2_ with/without substrates.

**Supplementary Fig. 6.** The standard curves of substrate and products.

**Supplementary Fig. 7.** **NMR spectra and calibration curves for quantification of EtOH and** **CH_3_COOH.** (a) ^1^H NMR spectra of EtOH and CH_3_COOH at various concentrations. (b) The calibration curves of EtOH. (c) The calibration curves of CH_3_COOH.

**Supplementary Fig. 8.** The effect of EtOH concentration on the reaction. Reaction conditions: Ni(OH)_2_ anode, Pt mesh cathode, 100 mM PhCH_2_OH, 5 mL 1.5 M K_2_CO_3_, 100 mA/cm^2^, 2 h, room temperature.

**Supplementary Fig. 9.** DFT-computed free energy profile (kcal/mol) for aldol reaction between benzaldehyde and acetaldehyde.

**Supplementary Fig. 10.** The hydration barrier of PhCHO and CH_3_CHO under neutral and alkaline conditions, and the BDFE of the hydrated PhCHO and the hydrated CH_3_CHO.

**Supplementary Fig. 11.** Mass spectra of commercial acetaldehyde and benzaldehyde after a spontaneous exchange in (a, c) 1 M KOH H_2_O solution and in (b, d) 1 M KOH D_2_O solution.

**Supplementary Fig. 12.** ^1^H NMR of a 0.01 M KOH with 10 mM benzaldehyde solution in D_2_O. The ratio of the integrations of the aldehyde peak at 6.52 with that of the aromatic peaks between 7.0 and 7.8 was compared to the expected value of 1:5 to determine that 23% (1-5/6.52*100%) of the benzaldehyde was in the diol form.

**Supplementary Fig. 13.** ^1^H NMR of a 0.1 M KOH with 10 mM benzaldehyde solution in D_2_O. The ratio of the integrations of the aldehyde peak at 9.38 with that of the aromatic peaks between 7.0 and 7.8 was compared to the expected value of 1:5 to determine that 47% (1-5/9.38*100%) of the benzaldehyde was in the diol formation.

**Supplementary Fig. 14.** DFT-computed free energy profile (kcal/mol) for nucleophilic addition and deprotonation of CH_3_CHO.

**Supplementary Fig. 15.** The conversion of EtOH and the selectivity of CH_3_COOH in the electrolyte with different pH value. Reaction conditions: Ni(OH)_2_ anode, Pt mesh cathode, 100 mM PhCH_2_OH, 600 mM EtOH, 5 mL electrolyte, 100 mA/cm^2^, 2 h, room temperature.

**Supplementary Fig. 16.** The effect of cation concentration on the reaction. Reaction conditions: Ni(OH)_2_ anode, Pt mesh cathode, 100 mM PhCH_2_OH, 5 mL electrolyte, 100 mA/cm^2^, 2 h, room temperature.

**Supplementary Fig. 17.** The conversion of EtOH and the selectivity of CH_3_COOH in the mix of KOH and K_2_CO_3_ solution. Reaction conditions: Ni(OH)_2_ anode, Pt mesh cathode, 100 mM PhCH_2_OH, 600 mM EtOH, 5 mL electrolyte, 100 mA/cm^2^, 2 h, room temperature.

**Supplementary Fig. 18.** Snapshots of PhCHO after 20 ns in the mix of aldehyde electrolyte system as a function of K_2_CO_3_ concentration.

**Supplementary Fig. 19.** Snapshots of CH_3_CHO after 20 ns in the mix of aldehyde electrolyte system as a function of K_2_CO_3_ concentration.

**Supplementary Fig. 20.** Radial distribution function (a) and the coordination number curves (b) for the PhCHO and H_2_O.

**Supplementary Fig. 21.** Radial distribution function (a) and the coordination number curves (b) for the CH_3_CHO and H_2_O.

**Supplementary Fig. 22.** Radial distribution function (a) and the coordination number curves (b) for the K^+^ ions and H_2_O.

**Supplementary Fig. 23.** The number of hydrogen bonds of (a) PhCHO and H_2_O (b) CH_3_CHO and H_2_O.

**Supplementary Fig. 24.** Nyquist plots of Ni(OH)_2_ and Pt-Ni(OH)_2_ electrode for (a, c) OER and (b, d) AOR.

**Supplementary Fig. 25.** Bode plots of Ni(OH)_2_ and Pt-Ni(OH)_2_ electrode for (a, c) OER and (b, d) AOR.

**Supplementary Fig. 26.** I-t curves of Ni(OH)_2_ and Pt-Ni(OH)_2_ electrode at different potentials.

**Supplementary Fig. 27.** The conversion of EtOH and the selectivity of CH_3_COOH over the different catalysts.

**Supplementary Fig. 28.** The GC-MS spectra of the reaction (a) using Pt and OD-Cu as the cathode in the mix of 0.4 M KOH, 1.0 M K_2_CO_3_ and 1.0 M KNO_3_ electrolyte; (b) MS of CHD; (c) in the divided cell using 100 mM CAL as the substrate in the mix of 0.4 M KOH and 1.5 M K_2_CO_3_ electrolyte. Other reaction conditions: 100 mA/cm^2^, 2 h, room temperature.

**Supplementary Fig. 29.** LSV curves of Pt in 0.4 M KOH with different concentrations of K_2_CO_3_

**Supplementary Fig. 30.** Reaction between (a) PhCHO and ammonia; (b) CAL and ammonia. Reaction conditions: 100 mM aldehyde, the mix of 0.4 M KOH and 1.5 M K_2_CO_3_ solution, 2 h, room temperature.

**Supplementary Fig. 31.** The products distribution of reaction between PhCH_2_OH and ammonia. Reaction conditions: 100 mM PhCH_2_OH, the mix of 0.4 M KOH and 1.5 M K_2_CO_3_ solution, 100 mA/cm^2^, 2 h, room temperature.

**Supplementary Fig. 32.** LSV curves of (a) Pt, (b) Ni, (c) Mo, (d) Ti, (e) Fe, (f) Ag, (g) Co, (h) Cu, and (i) Cu/Cu plate in 0.4 M KOH + 1.5 M K_2_CO_3_ (black line), with 10 mM CAL (red line), 10 mM CAL + 100 mM KNO_3_ (blue line).

**Supplementary Fig. 33.** E-t curves in the different electrolysis system. | represent undivided cell; || represent divided cell.

**Supplementary Fig. 34.** The effect of the different temperatures on the reaction.

**Supplementary Fig. 35.** The conversion of EtOH and the selectivity of CH_3_COOH at different current density.

**Supplementary Fig. 36.** (a) UV-Vis absorption spectra of various NH_3_ concentrations and (b) calibration curve for quantify NH_3_.

**Supplementary Fig. 37.** Stability test of the reaction.

**Supplementary Fig. 38.** *E-t* curves of stability test.


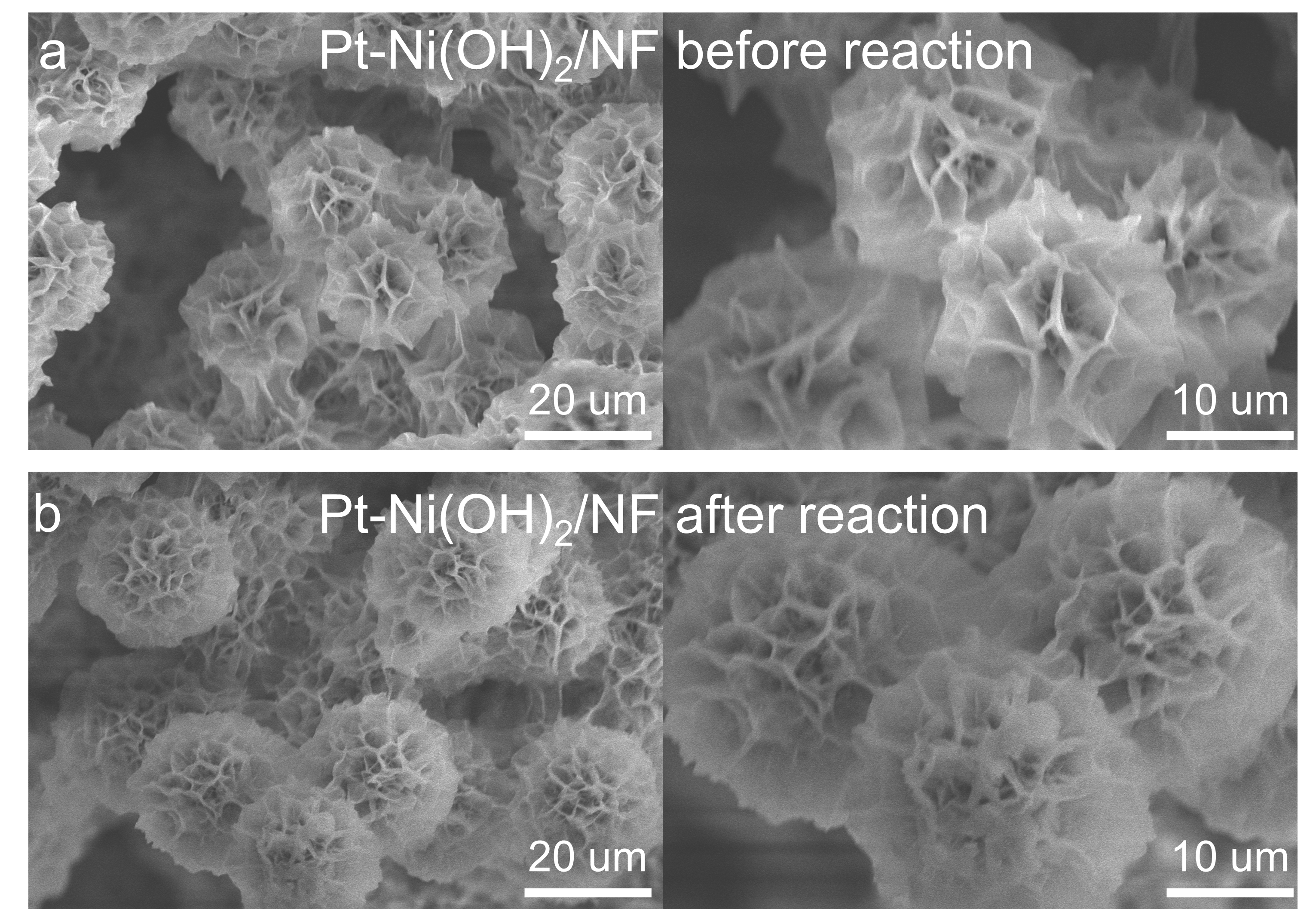


**Supplementary Fig. 39.** SEM images of Pt-Ni(OH)_2_ (a) before reaction; (b) after stability tests.

**Supplementary Fig. 40.** XRD pattern for Pt-Ni(OH)_2_/NF before and after stability tests.

**Supplementary Fig. 41.** XPS spectra of Pt-Ni(OH)_2_ before and after stability tests (a) Ni 2p region; (b) Pt 4f region.

**Supplementary Fig. 42.** Characterization of electrolyte after reaction. (a) ICP-MS of the electrolyte after cyclic stability test; (b) the pH value before and after stability tests.

**Supplementary Fig. 43.** The bond dissociation free energy of different aromatic alcohol.

**Supplementary Fig. 44.** The free energy barrier of condensation reaction for the different aromatic alcohol.

**Supplementary Tab. 1.** The pH value of electrolytes

| Electrolytes | pH value | Electrolytes | pH value |
| --- | --- | --- | --- |
| 1.0 M K_2_CO_3_+0.1 M KOH | 13.05 | 1.5 M K_2_CO_3_+0.1 M KOH | 13.16 |
| 1.0 M K_2_CO_3_+0.2 M KOH | 13.33 | 1.5 M K_2_CO_3_+0.2 M KOH | 13.45 |
| 1.0 M K_2_CO_3_+0.3 M KOH | 13.49 | 1.5 M K_2_CO_3_+0.3 M KOH | 13.61 |
| 1.0 M K_2_CO_3_+0.4 M KOH | 13.62 | 1.5 M K_2_CO_3_+0.4 M KOH | 13.72 |
| 1.0 M K_2_CO_3_+0.5 M KOH | 13.66 | 1.5 M K_2_CO_3_+0.5 M KOH | 13.79 |

**Supplementary Tab. 2.** The DFT-calculated free energy barrier for the synthesis of BA

| In the alkaline solution | ΔG (kcal/mol) | In the neutral solution | ΔG (kcal/mol) |
| --- | --- | --- | --- |
| b1→TSb3 | 14.2 | b1→TSb3 | 67.3 |
| b4→TSc1 | 10.2 |  |  |
| c2→TSc2 | 11.9 |  |  |
| c3→TSc3 | 13.9 |  |  |

**Supplementary Tab. 3.** The DFT-calculated free energy barrier for the hydration of aldehydes

| In the alkaline solution | ΔG (kcal/mol) | In the neutral solution | ΔG (kcal/mol) |
| --- | --- | --- | --- |
| PhCHO | 10.8 | PhCHO | 45.6 |
| CH_3_CHO | 10.4 | CH_3_CHO | 43.5 |

**NMR spectroscopy**


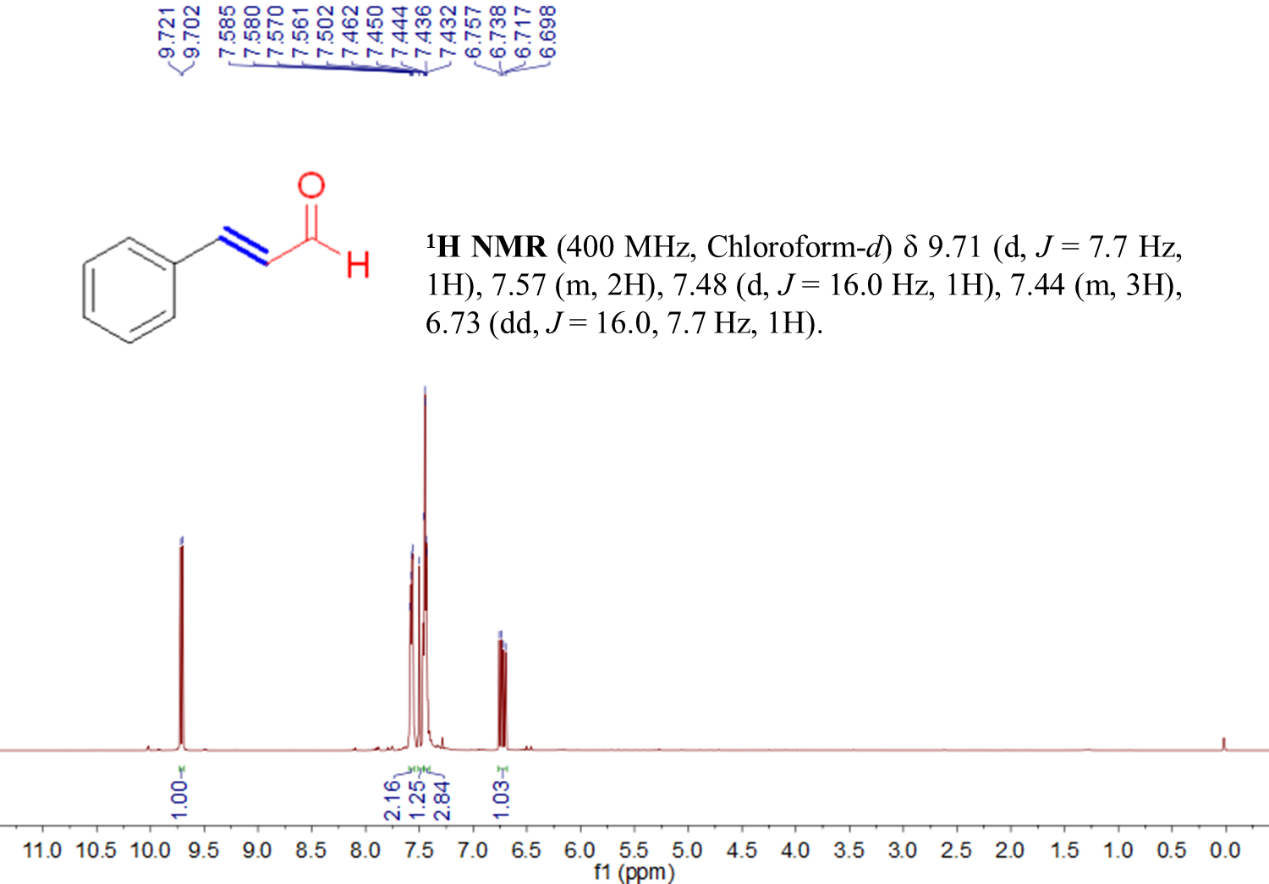


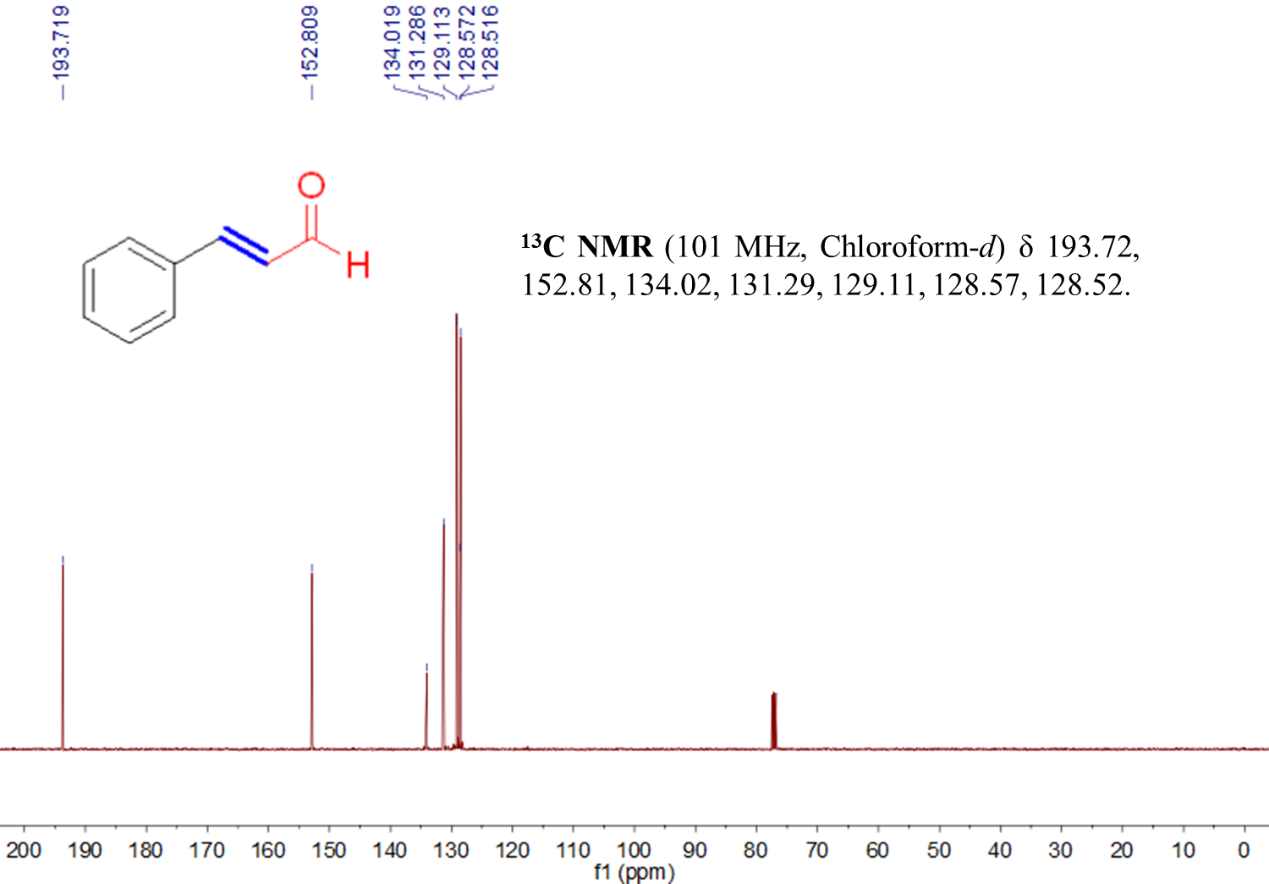


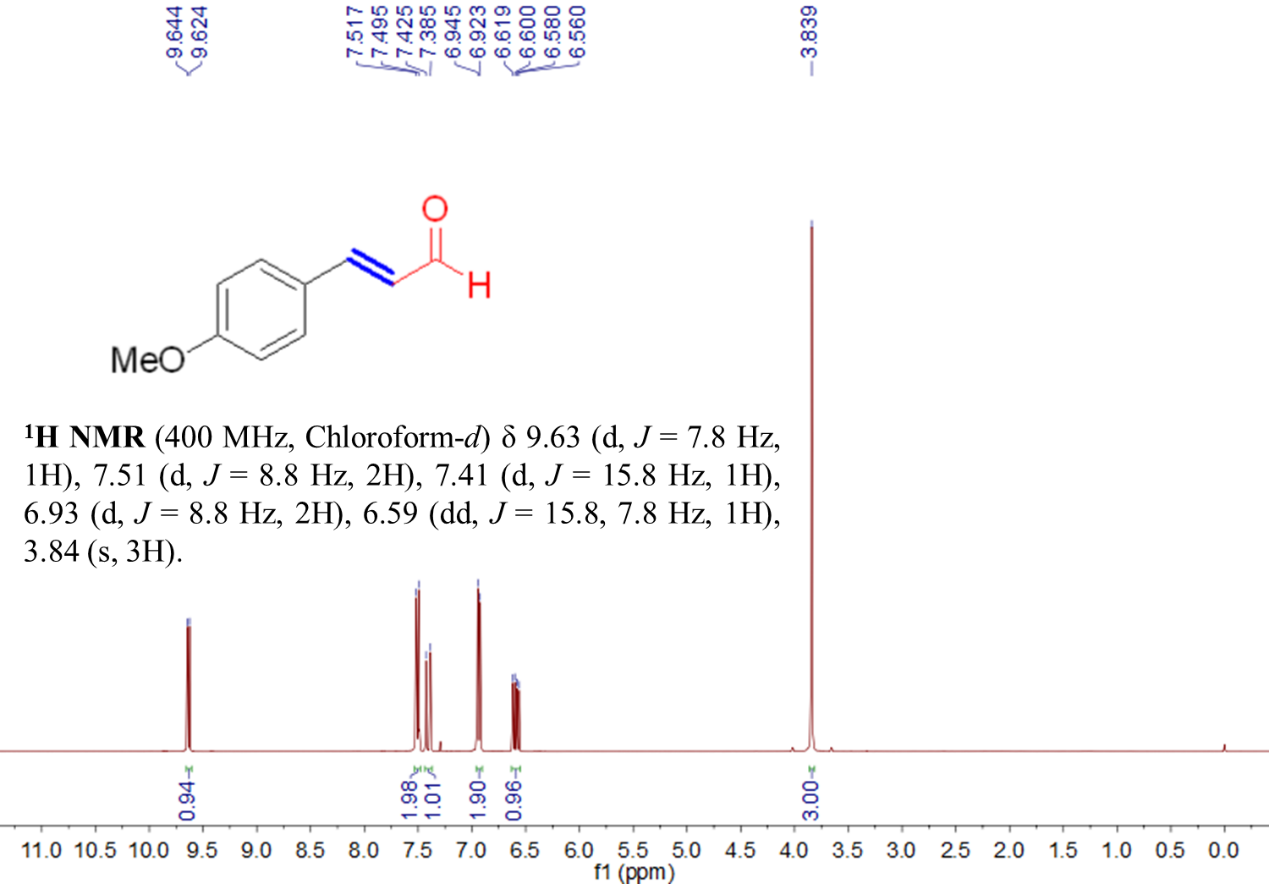


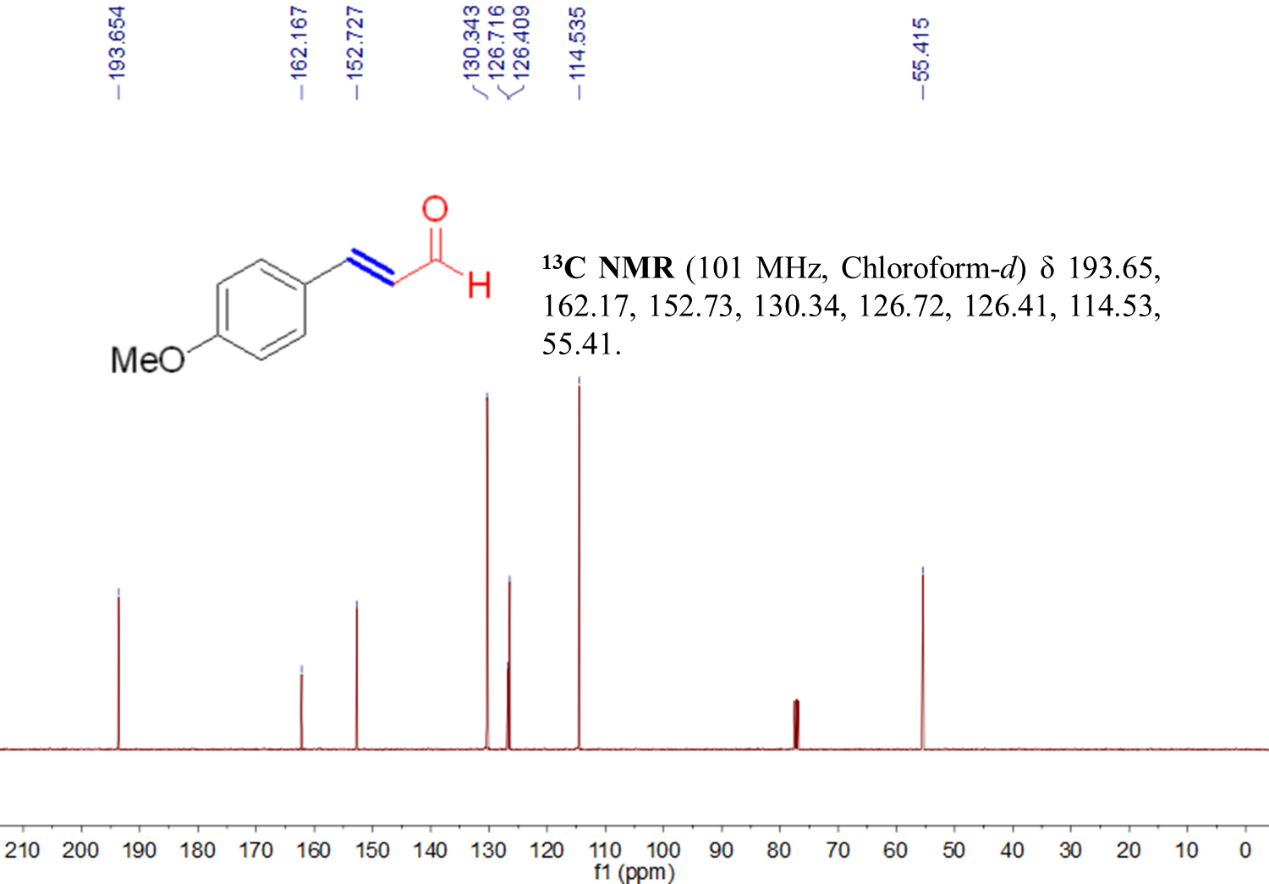


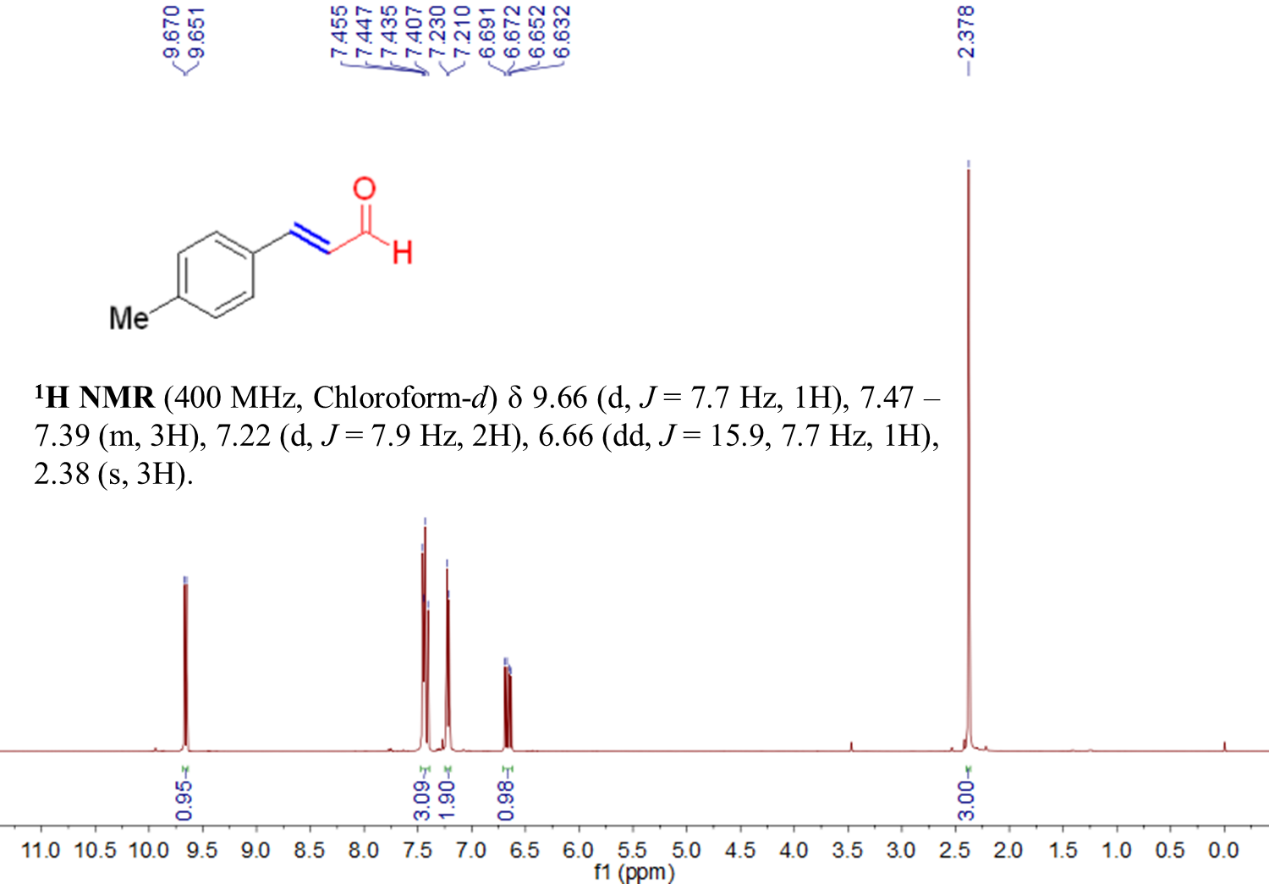


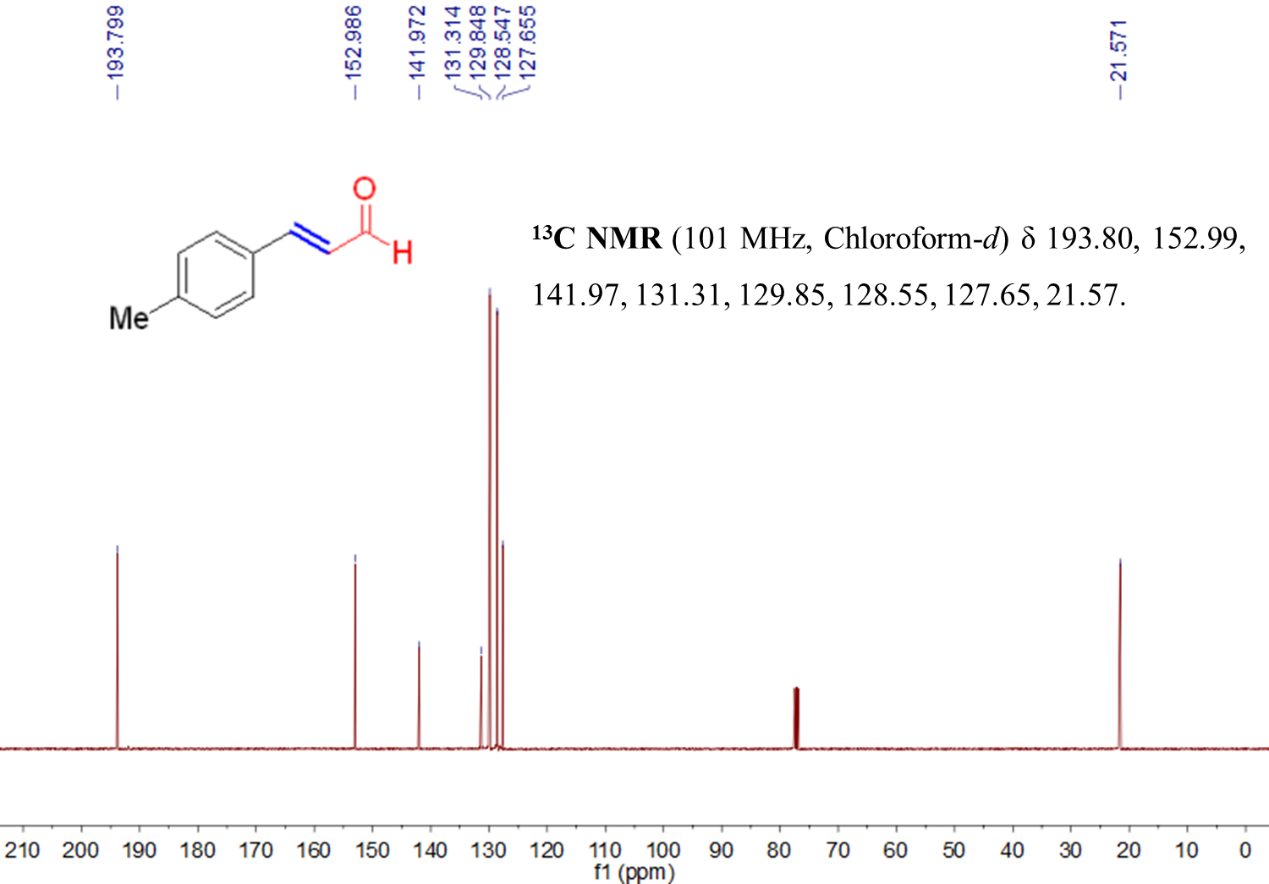


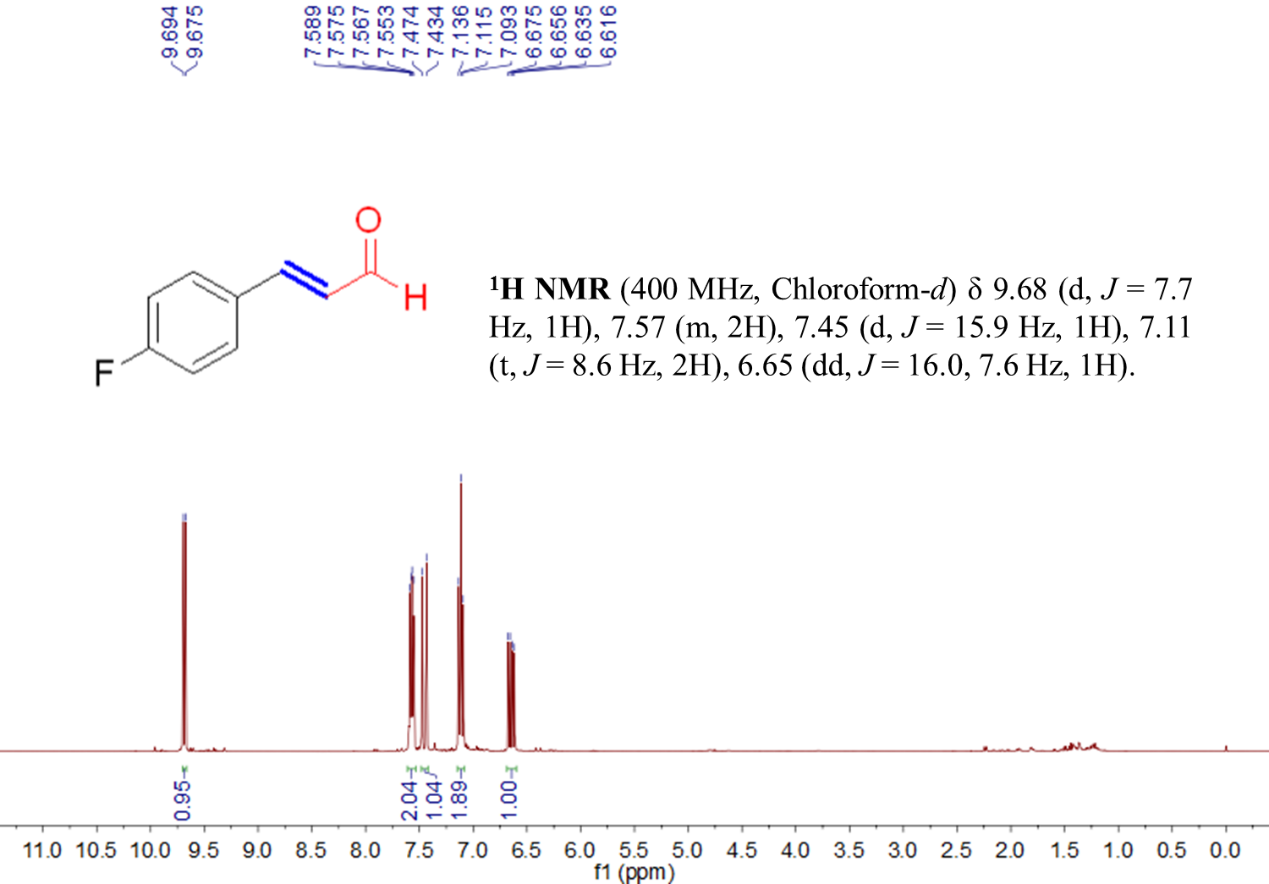


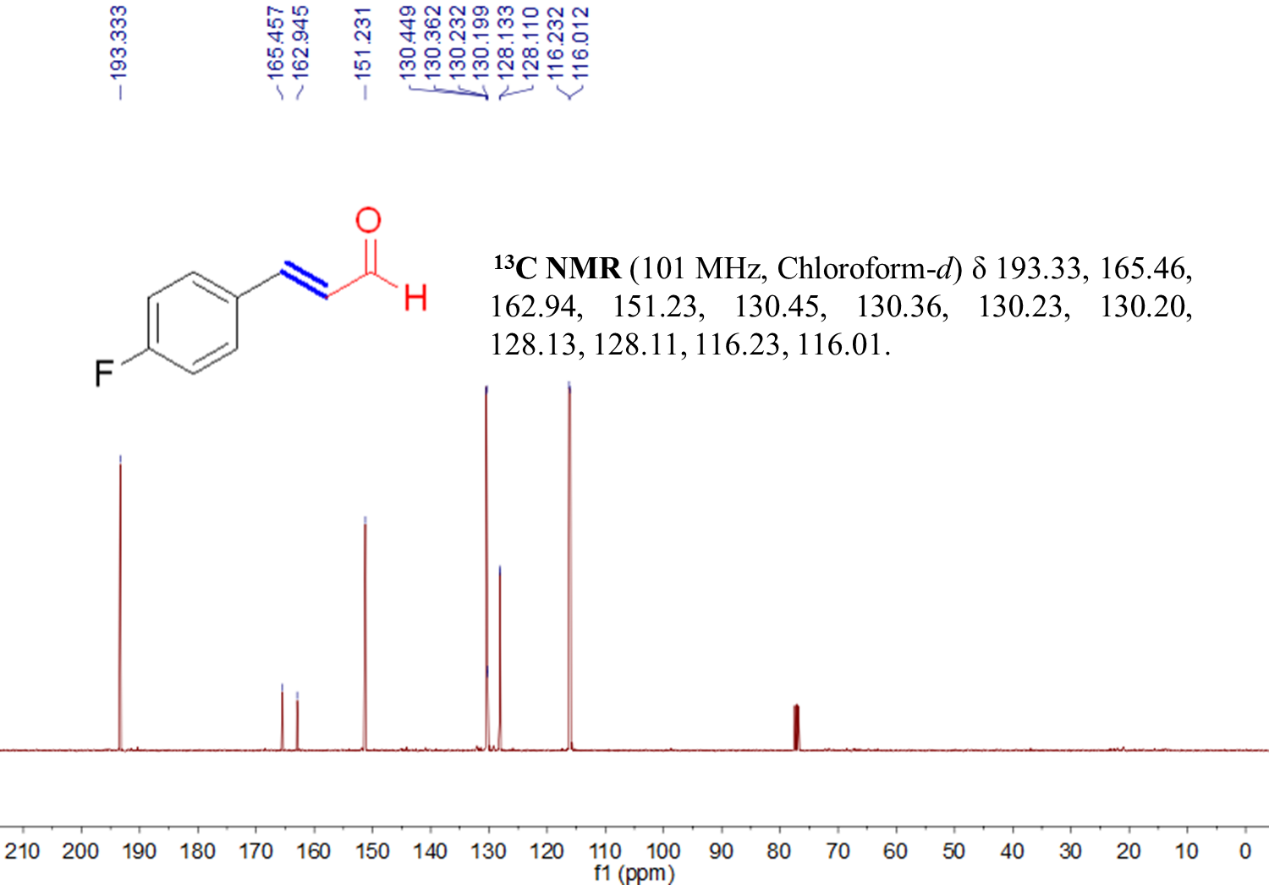


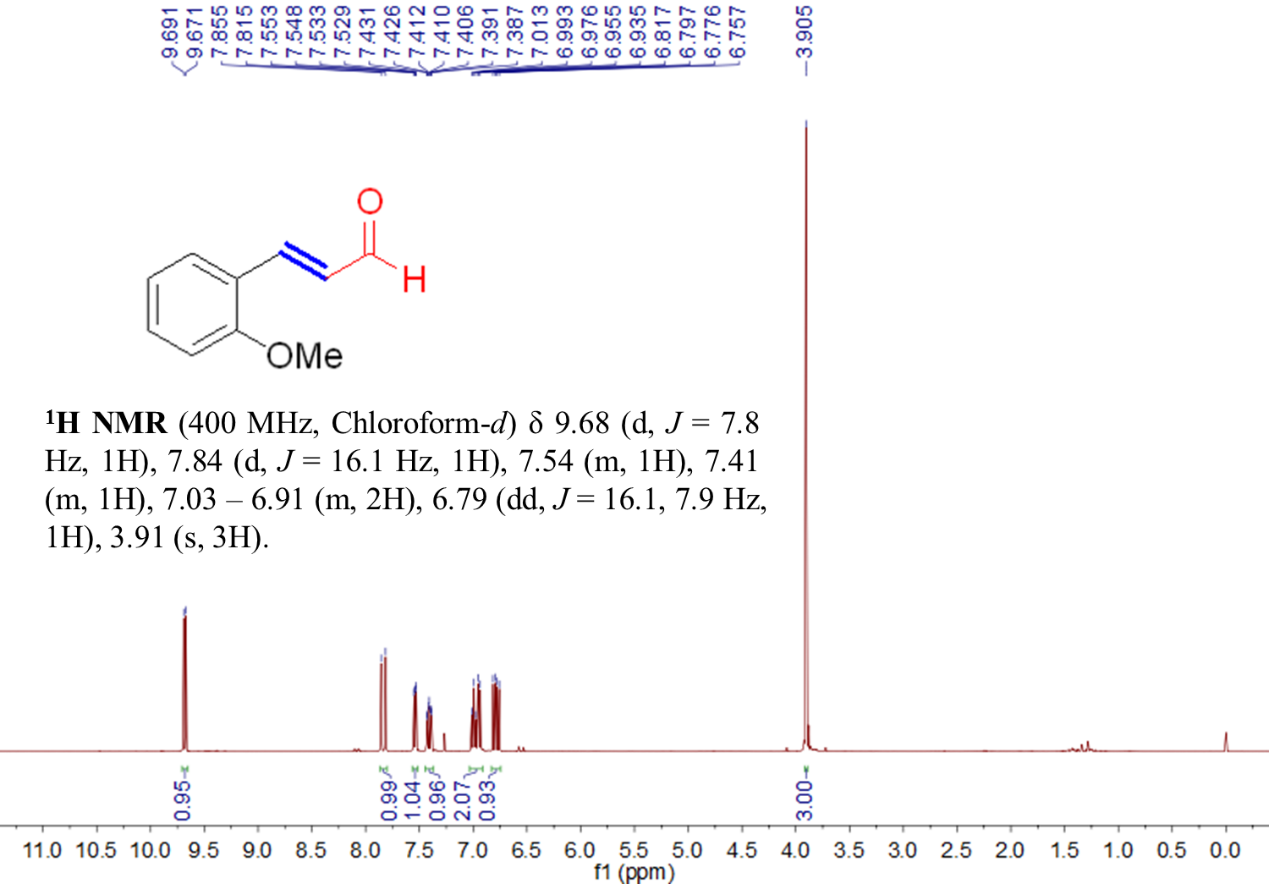


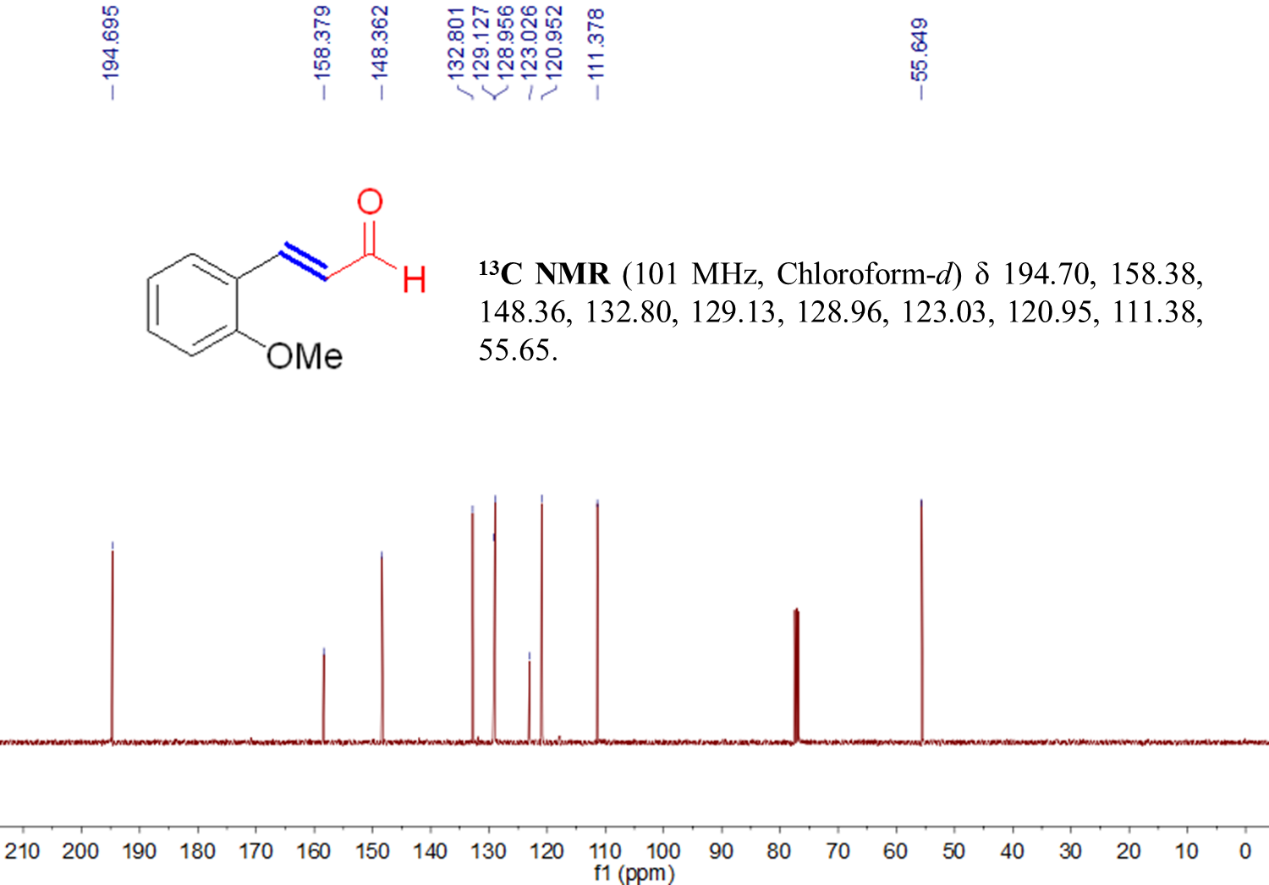

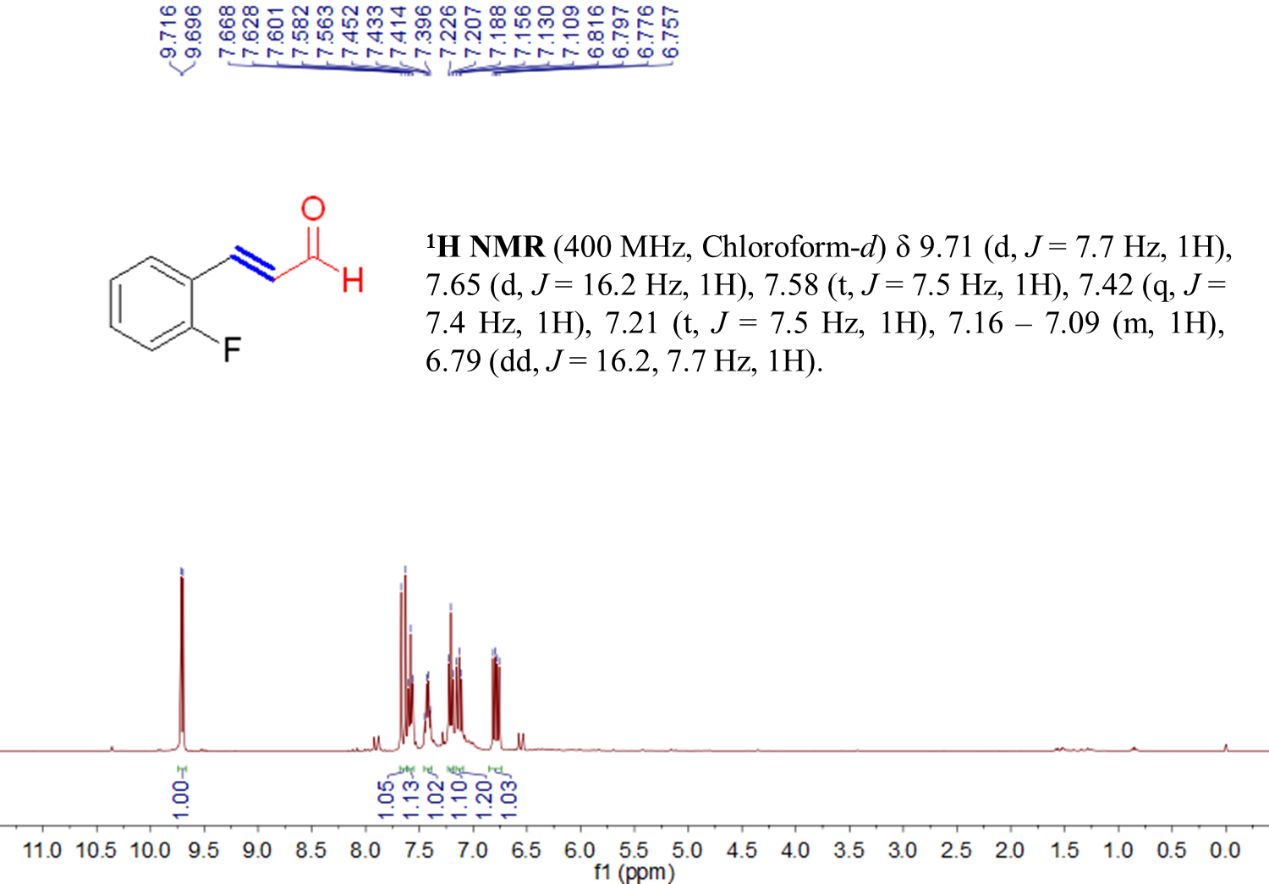


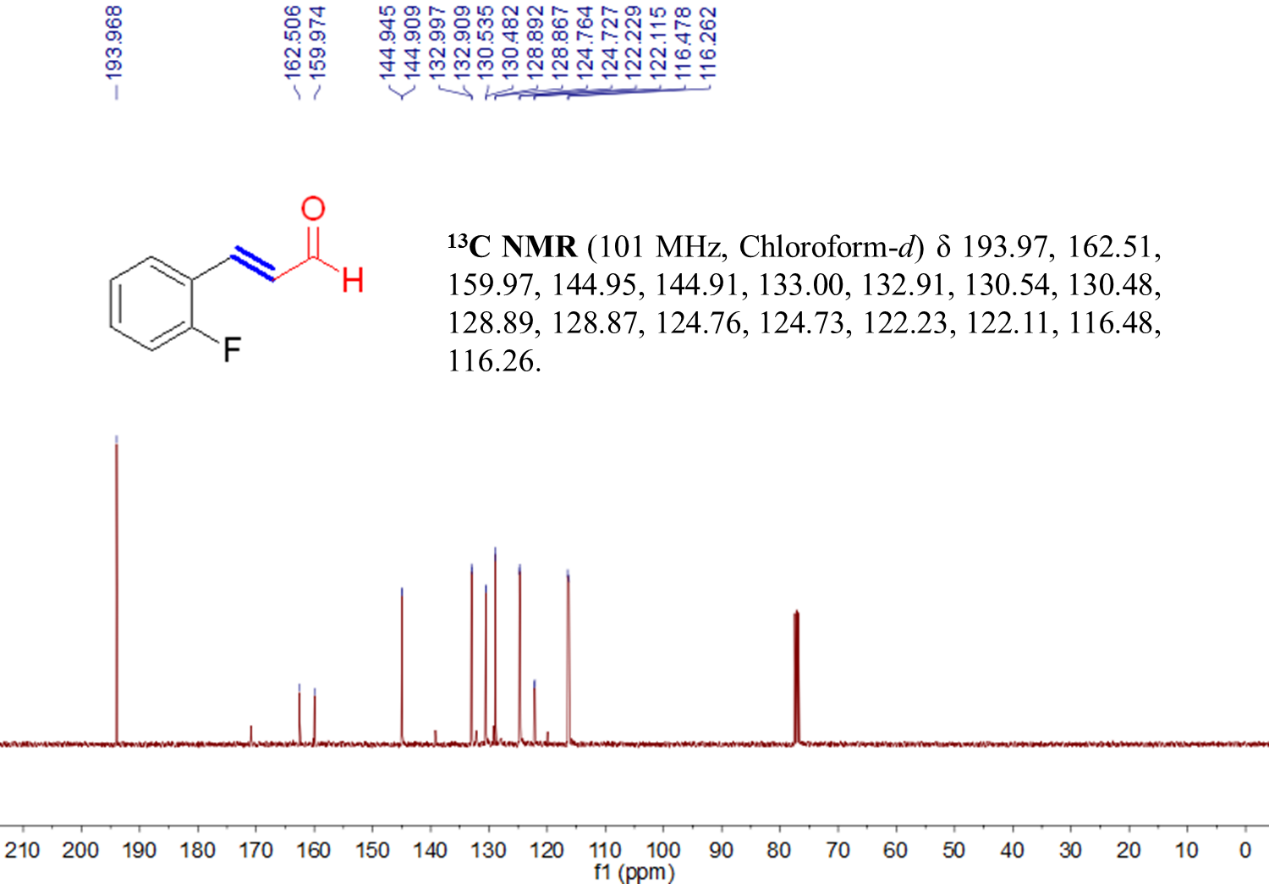


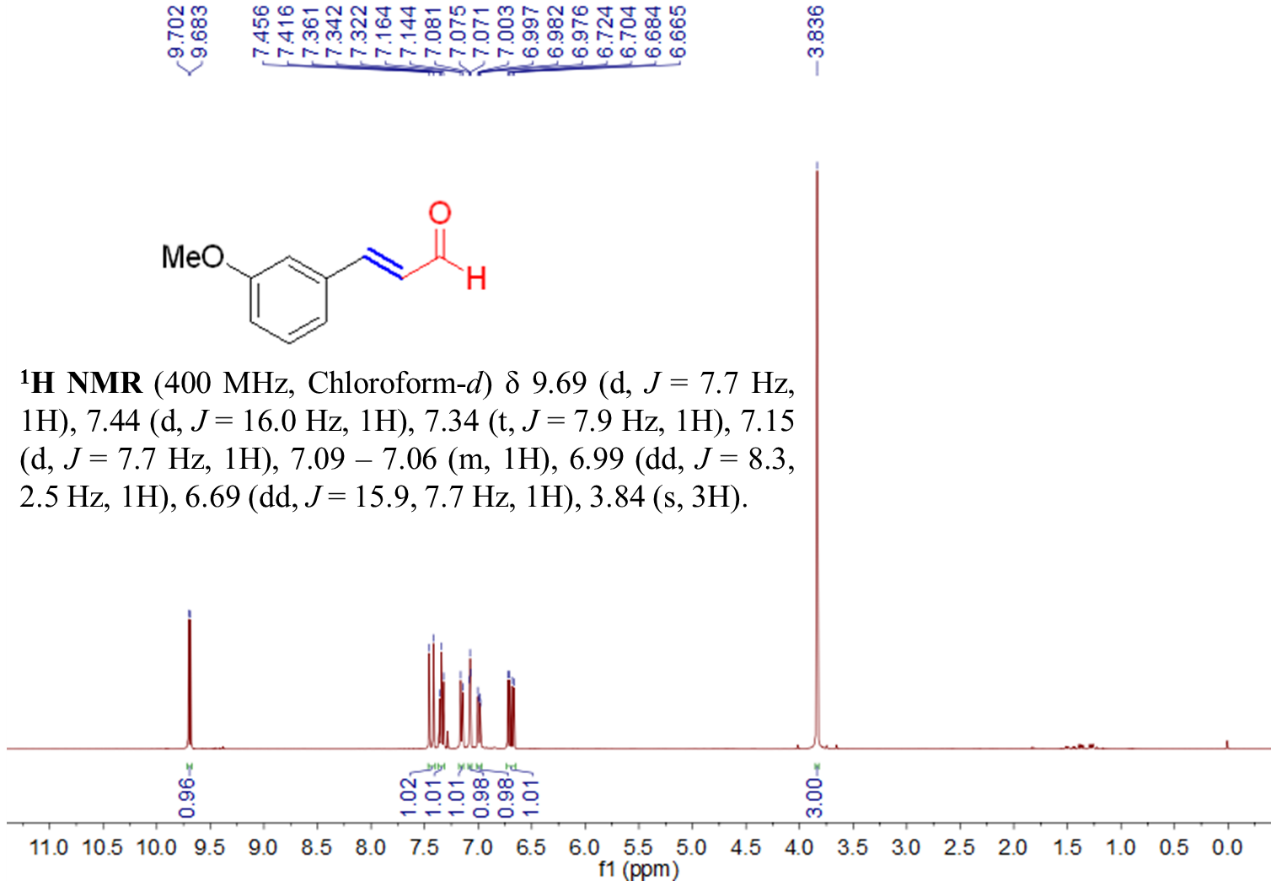


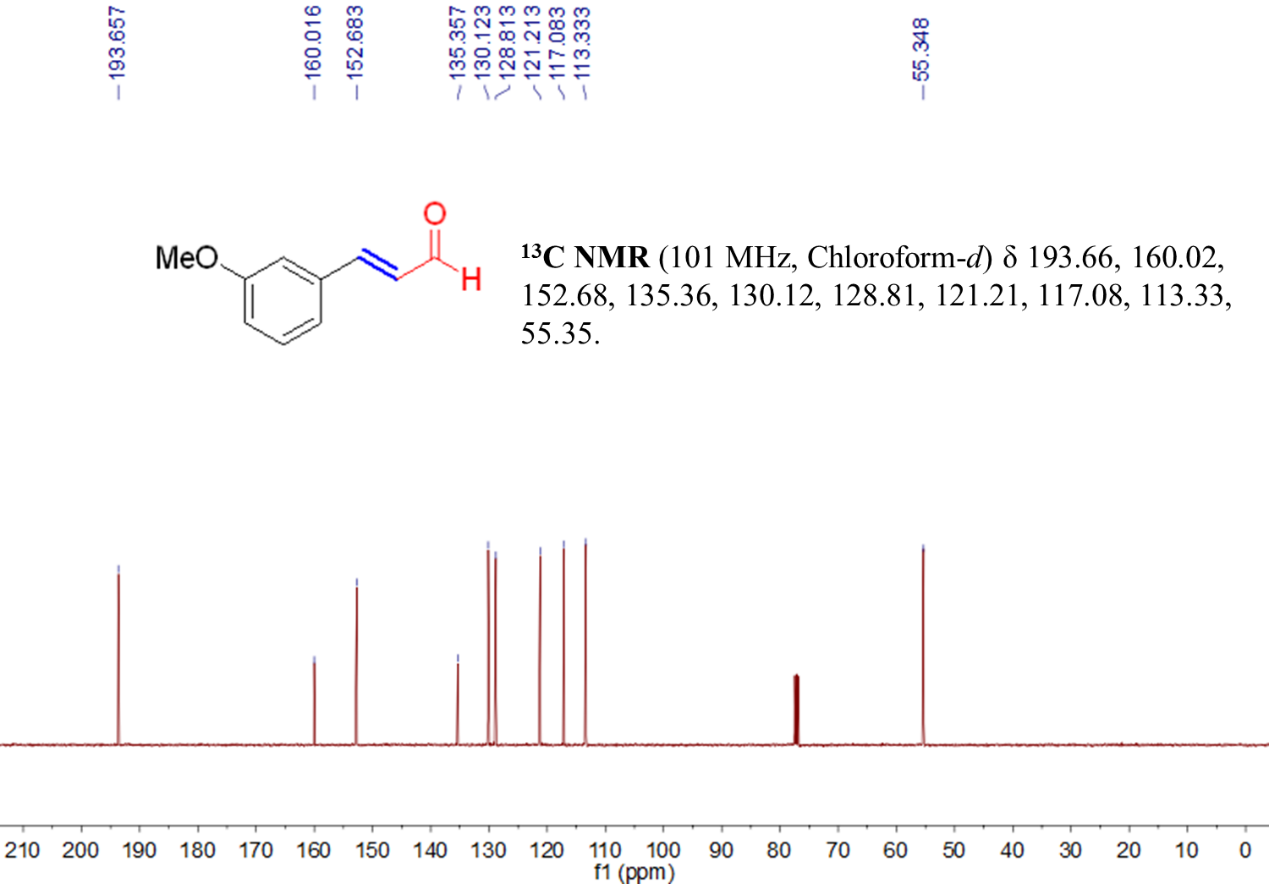


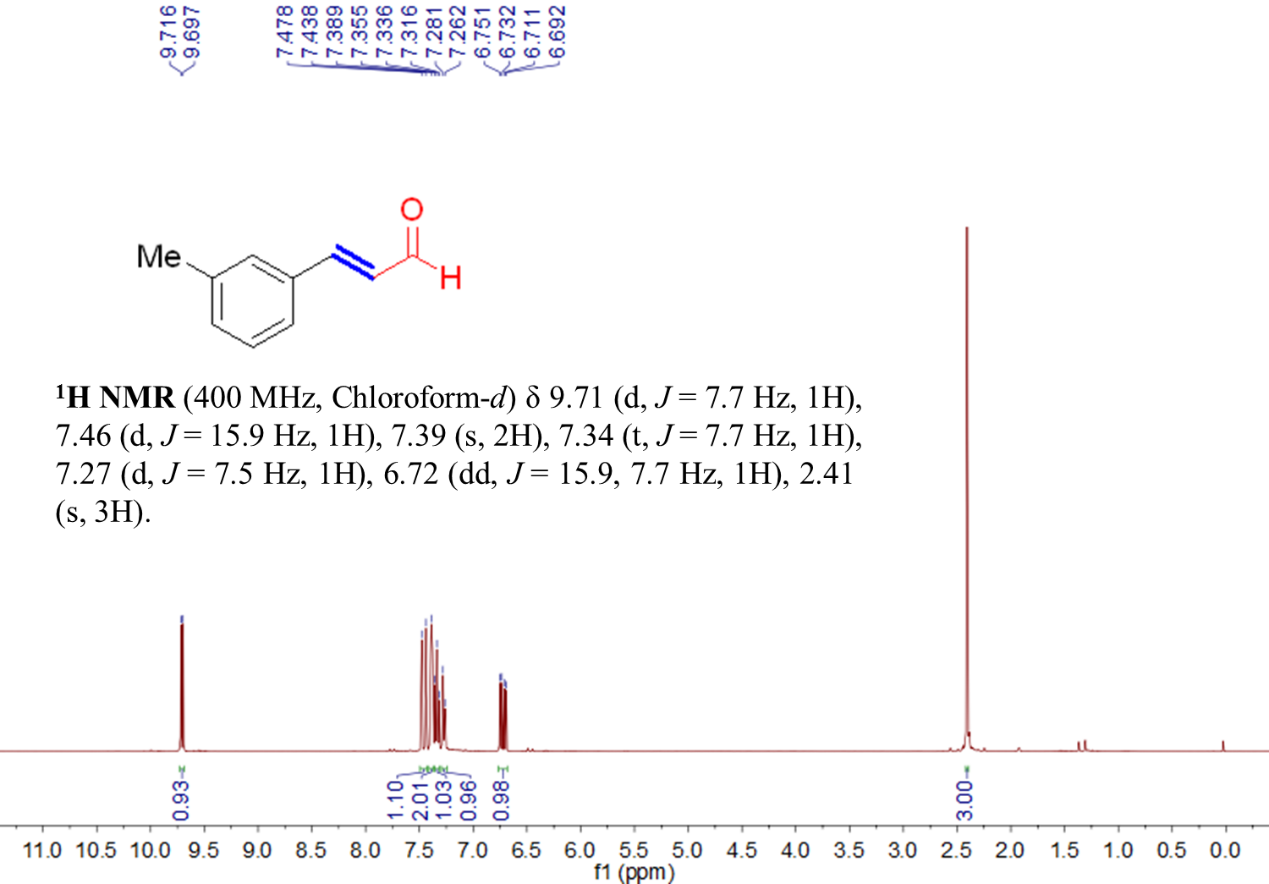


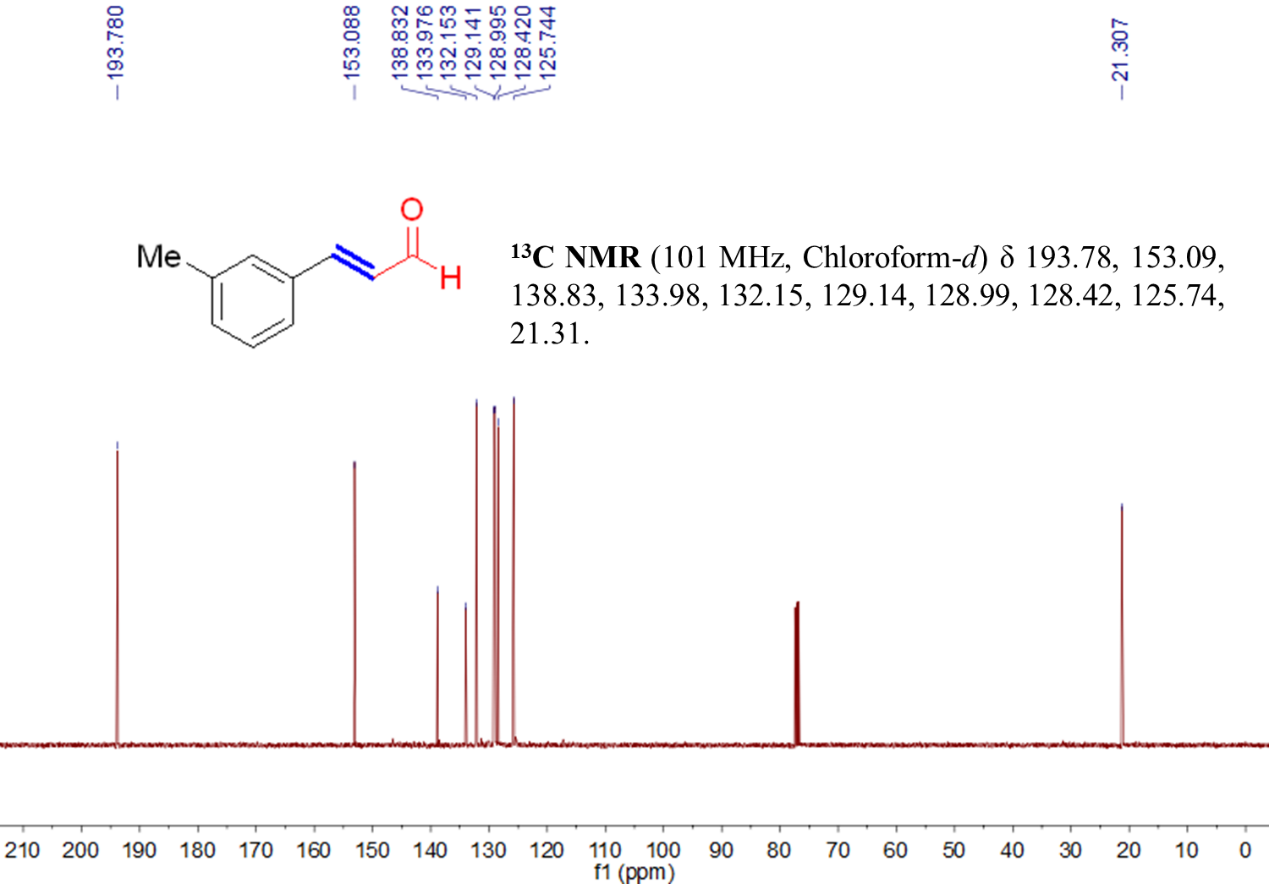


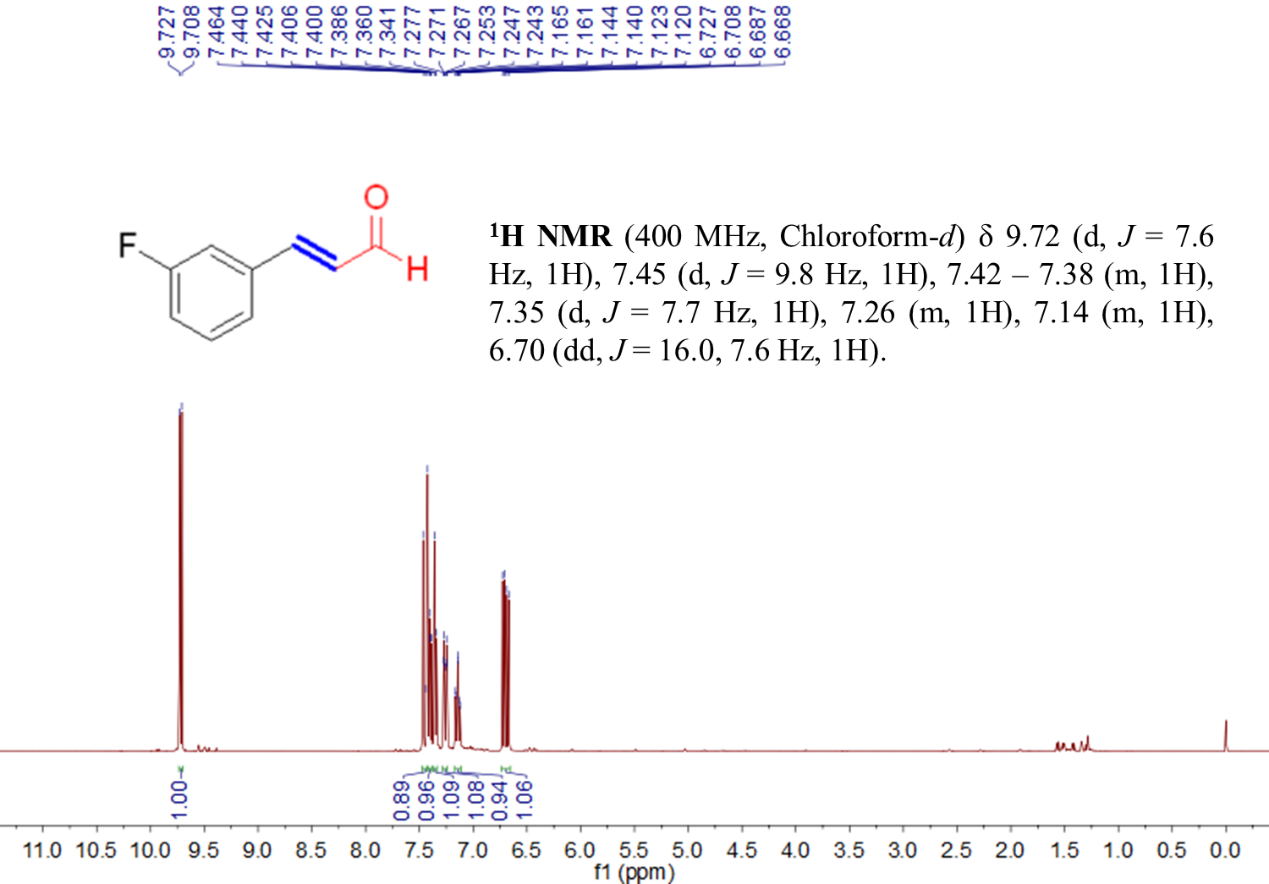


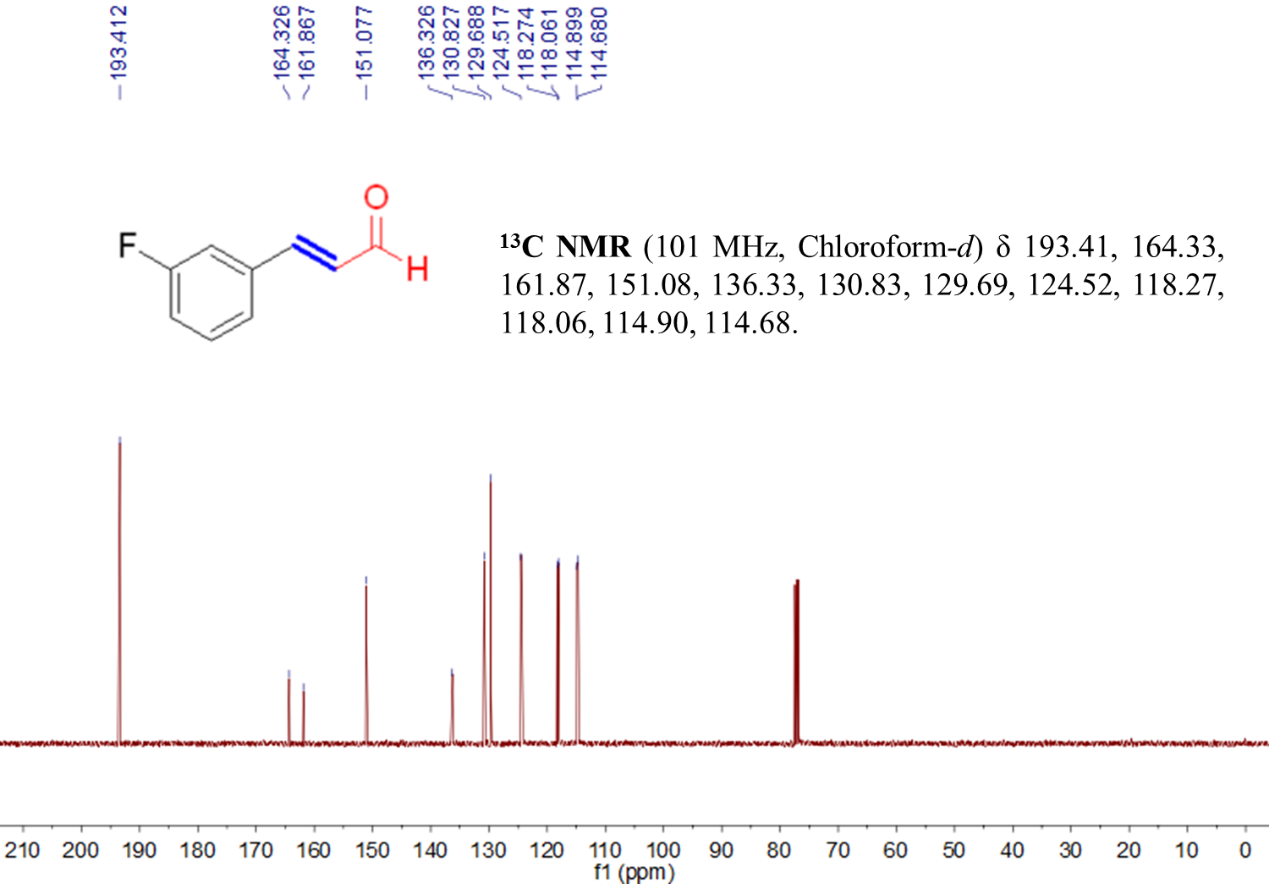


**Reference**

1. Zhao Y and Truhlar D. The M06 suite of density functionals for main group thermochemistry, thermochemical kinetics, noncovalent interactions, excited states, and transition elements: two new functionals and systematic testing of four M06-class functionals and 12 other functionals. *Theor Chem Acc* 2008; **120**: 215-241.

2. Rassolov V, Ratner M, Pople J *et al.* 6-31G* basis set for third-row atoms. *J Comput Chem* 2001; **22**: 976-984.

3. Weigend F. Accurate Coulomb-fitting basis sets for H to Rn. *Phys Chem Chem Phys* 2006; **8**: 1057-1065.

4. Marenich A, Cramer C and Truhlar D. Universal solvation model based on solute electron density and on a continuum model of the solvent defined by the bulk dielectric constant and atomic surface tensions. *J Phys Chem B* 2009; **113**: 6378-6396.

5. Frisch M, Trucks G, Schlegel H *et al.* Gaussian 16 Rev. C.01. Wallingford, CT; 2016.

6. Berendsen H, Grigera J and Straatsma T. The missing term in effective pair potentials. *J Phys Chem* 1987; **91**: 6269-6271.

7. Potoff J and Siepmann J. Vapor–liquid equilibria of mixtures containing alkanes, carbon dioxide, and nitrogen. *AIChE Journal* 2001; **47**: 1676-1682.

8. Chowdhuri S and Chandra A. Hydration structure and diffusion of ions in supercooled water: Ion size effects. *J Chem Phys* 2003; **118**: 9719-9725.

9. Lu T and Chen F. Multiwfn: A multifunctional wavefunction analyzer. *J Comput Chem* 2012; **33**: 580-592.

10. Lennard-Jones J. Cohesion. *Proc Phys Soc* 1931; **43**: 461-482.

11. Lorentz H. Ueber die anwendung des satzes vom virial in der kinetischen theorie der gase. *Ann Phys* 1881; **248:** 127-136.

12. Berthelot D, Sur le mélange des gaz C, Hebd. Seances *Acad Sci* 1989; **126:** 1703-1855.

13. Bouazizi S and Nasr S. Local order in aqueous lithium chloride solutions as studied by X-ray scattering and molecular dynamics simulations. *J. Mol. Struct.* 2007; **837:** 206-213.

14. Du H, Rasaiah J, Miller J. Structural and dynamic properties of concentrated alkali halide solutions:  A molecular dynamics simulation study. *J Phys Chem B* 2007; **111:** 209-217.
